# Individual changes in stress, depression, anxiety, pathological worry, posttraumatic stress, and health anxiety from before to during the COVID-19 pandemic in adults from Southeastern Germany

**DOI:** 10.1186/s12888-022-04148-y

**Published:** 2022-08-05

**Authors:** Theresa F. Wechsler, Melissa Schmidmeier, Stefanie Biehl, Jennifer Gerczuk, Fiorella-Maria Guerrero-Cerda, Andreas Mühlberger

**Affiliations:** Department of Psychology, Clinical Psychology and Psychotherapy, University of Regensburg, Regensburg, Germany

**Keywords:** COVID-19, Mental health, Stress, Depression, Anxiety, Panic disorder, Generalized anxiety, Health anxiety, Pathological worry, Posttraumatic stress

## Abstract

**Background:**

Many studies have previously compared the prevalence or sample means of distress and mental health problems from before to during the COVID-19 pandemic, while results on changes at the individual-level, and regarding multiple outcome measures are demanded.

**Methods:**

This online study investigated individual changes in stress and mental health from before the COVID-19 pandemic to the first lockdown in adults from Southeastern Germany. This region was selected as it was where SARS-CoV-2 was first documented in Germany, and also due to the implementation of strict stay-at-home orders and social contact prohibitions. From April 10–27, 2020, we collected state measures and their clinical relevance for the subareas of perceived stress: worries, tension, joy, and demands. We also collected information regarding the following mental health problems: depression, anxiety, pathological worry, posttraumatic stress disorder (PTSD), and health anxiety; as well as retrospective measures of how participants felt they have changed in comparison to before the pandemic, ranging from worse to better.

**Results:**

The analytical sample comprised 396 adult participants. On average, participants experienced increases in worries, tension, and lack of joy, and increases in mental health problems, but a decrease in demands. Perceived increases in symptoms of depression (26.0%) and PTSD (25.5%) were significantly more frequent than in symptoms of anxiety (particularly acute fear and panic) (5.6%), pathological worry (9.8%), and health anxiety (7.3%) (*ps*<.001). One per 10 participants (10.4%) reported an increase in depressive symptoms, and nearly two per 10 (18.4%) an increase in PTSD symptoms and additionally showed a clinically relevant symptom strain during lockdown. Interestingly, mainly non-specific PTSD symptoms associated with a general stress reaction were experienced to be increased.

**Conclusion:**

The findings suggest a dissociation of perceived changes in subareas of stress and mental health with a particular experience of increases in depressive and general stress symptoms and a decrease in external demands. This points to a need for a more differentiated view on the impact of the COVID-19 pandemic on stress and mental health, and for targeted interventions for mental health problems arising frequently during the pandemic.

**Supplementary Information:**

The online version contains supplementary material available at 10.1186/s12888-022-04148-y.

## Background

### SARS-CoV-2 cases and protection measures during the early phase of the COVID-19 pandemic in Germany

**Fig. 1:**
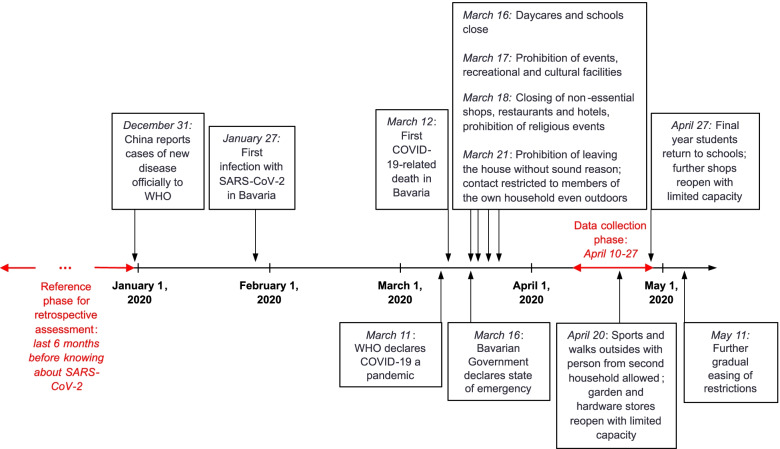
Milestone dates of the early phase of the COVID-19 pandemic and Bavarian lockdown measures. The figure reports milestone dates concerning the spreading of SARS-CoV-2 and the early phase of the COVID-19 pandemic [1, 2], and concerning lockdown measures in Bavaria [3–5, 7–9, 11, 12]. Between April 10–27, 2020 (recruitment and data collection phase), state measures of stress and mental health problems during lockdown, as well as retrospective measures comparing the current state to the last six months before knowing about SARS-CoV-2 were conducted

The first German SARS-CoV-2 case was registered in Southeastern Germany (Bavaria) on January 27, 2020 [1]. During March and April 2020, the cumulative number of SARS-CoV-2 cases and deaths with COVID-19 increased continuously, whereat approximately one fourth of all German cases were registered in Bavaria [2] (Additional Figure 1). From March 16, 2020, the Bavarian government declared protection measures to combat the spreading of the virus [3–5] (Fig. 1). As a particularly strict regulation, starting March 21, 2020, it was prohibited to meet members of another household even outdoors, and to leave the house without sound reason [4]. On March 22, 2020, the Federal German Government also communicated protection measures for the whole of Germany. These included leaving the house only for a valid reason, however, they allowed meeting outdoors with one person from another household [6]. Hence, the Bavarian lockdown was the strictest within Germany. Starting April 20, 2020, a first easing of lockdown measures was implemented in Bavaria, and starting April 27, 2020, the Bavarian government implemented broader openings [7–9] (Fig. 1). For the whole of Germany, lockdown openings were implemented starting May 4, 2020 [10].

### Changes in psychological distress and mental health from before to during the early phase of the COVID-19 pandemic

Meta-analyses on cross sectional studies examining changes in stress and mental health in adults from the general population of different countries worldwide during the early phase of the COVID-19 pandemic mainly found increases in depression [13–16] and anxiety [14–16] compared to prepandemic prevalence rates. One meta-analysis also reported significant increases of posttraumatic stress disorder (PTSD) [14]. Another meta-analysis of longitudinal studies and natural experiments (studies comparing participants who were in lockdown with those who did not have such restrictions) found small but significant effects of lockdown for increases in depression and anxiety, and no significant effect for general distress [17]. A further meta-analysis of longitudinal cohort-studies specifically showed a larger increase in depression than in anxiety, also no significant increase for distress, and additionally no significant changes in non-specific mental health measures, well-being, and other mental health problems, and a significant decrease for symptoms of psychosis [18]. To the best of our knowledge, no meta-analyses on changes in pathological worrying and health anxiety from before to the early phase of the COVID-19 pandemic have been published so far.

Original studies examining changes from before to during the early phase of the COVID-19 pandemic specifically for the German general population, found increased distress [19, 20], depression [19–23], anxiety [19–24], and health anxiety [25, 26]. Other studies, in contrast, found no significant changes in stress [27] and overall mental health [27, 28], or even showed a significant decrease in daily hassles [29] and mental health problems [29, 30]. Concerning PTSD symptoms in the German general population, one study found that 15% of their respondents met the cut-off for COVID-19 related traumatic distress [28], while results on changes in PTSD symptoms from before to during the pandemic have not been reported yet. For changes in pathological worrying in the general population, we did not find results from previous studies. Also, for stress and mental health during the COVID-19 pandemic among the general population of the Bavarian region, no results have been published until now. One exception is the preprint of a study comparing the severity of depressive symptoms during the late phase of the first COVID-19 lockdown in adults from the German federal states of Bavaria with adults from Lower Saxony [31]. However, changes in depressive symptoms compared to before the pandemic were not examined for the Bavarian subsample.

### Changes at the level of the individual

It has to be considered that the reported original studies and meta-analyses on changes in stress and mental health problems during the early phase of the COVID-19 pandemic mainly compared prevalence rates of elevated stress or symptoms [13–16, 18–20, 22, 24, 27] or sample means concerning stress or symptom severity [17, 18, 20, 21, 23, 25, 26, 27, 29] during and before the COVID-19 pandemic, derived from different samples [13–16, 19, 21–25, 27, 30], or assessed retrospectively [20, 26] or longitudinally [17, 18, 28, 29] within the same sample. Findings on changes in distress and mental health at the level of the individual, in contrast, are still rare. However, they are relevant as sample mean and prevalence comparisons do not consider individual increases and decreases. The few examples used different approaches of individual-level change assessment. Longitudinal studies assessed the same outcome measure before and after the COVID-19 outbreak [32, 33], or from before first lockdown (but after the COVID-19 outbreak) to during first lockdown [28, 29]. Retrospective studies assessed *individually perceived changes* by asking participants during the pandemic to report on how they feel they have changed after the COVID-19 outbreak in comparison to before, ranging from worse, or more specifically an increase of symptoms, to better, or more specifically a decrease of symptoms [34, 35].

Focusing on individual-level changes from before the pandemic to the early phase of the pandemic, one longitudinal study on the Dutch population provides frequencies on changes in preexisting moderate to high anxiety and depression symptoms from November 2019 to the earlier phase of the pandemic in March 2020, showing a remission in 16.1%, an improvement in 5.4%, no change in 54.3%, and a worsening in 24.2% [32]. Another longitudinal study from the UK showed that 28.6% of the participating adults without a common mental disorder less than one year before reached the cut-off for one in April 2020 during the COVID-19 pandemic, and that a recovery from a common mental disorder assessed before the pandemic was found in 38.4% [33]. An Australian study retrospectively asked adults from the general population about changes in their mental health within one single item, and found that their mental health status since the outbreak of the pandemic was a little worse in 55.1% of all participants, a lot worse in 22.9%, a little better in 3.8%, and a lot better in 0.7% [34]. A further retrospective study on the German and UK population assessed subjective changes in mental health during the pandemic in comparison to before, and found that 22.8% of the German participants reported an increase, 2.5% a decrease, and 71.7% the same amount of mental health symptoms, while 27.0% of the UK participants reported an increase, 3.6% a decrease, and 64.1% no change [35]. However, none of the mentioned studies on changes at the level of the individual discriminated between specific mental health syndromes like depression or anxiety, and therefore, no comparisons between the extent of individual changes in different subareas of stress and mental health could be examined until now.

### Research questions

Altogether, only few studies explored changes in stress and mental health from before to the early phase of the COVID-19 pandemic at the level of the individual, and they were restricted to unspecific outcome measures. The aim of this study was to explore how adults from Southeastern Germany (as the region with the strictest protection measures within Germany during the early phase of the COVID-19 pandemic) feel their stress and mental health has changed during the first lockdown after the COVID-19 outbreak, and thereby examine individually perceived changes in multiple outcome measures for perceived stress and mental health problems. The main research questions are: (1) During first lockdown in comparison to before the COVID-19 outbreak, do adults from Southeastern Germany perceive an increase, no change, or a decrease in the subareas of perceived stress worries, tension, joy, and demands, and in the mental health problems depression, anxiety, pathological worry, posttraumatic stress, and health anxiety? (2) Are there differences in the direction, extent, and frequency of individually perceived changes between areas of stress and mental health problems? (3) Do perceived increases in stress and mental health problems from before the COVID-19 outbreak to the first lockdown go along with a clinically relevant stress level and symptom severity during lockdown? Overall, mainly the individual feeling of being worse, but also the feeling of being better or unchanged after the outbreak was explored. As exploratory question, we examined the influence of sex and age on changes in stress and mental health, and on increases going along with a clinically relevant stress or symptom strain during lockdown. Concerning age, we were especially interested in the examination of differences between older participants (>50 years) with a higher risk for a severe illness with COVID-19 according to the Robert-Koch-Institute [36], and younger participants with a lower risk. Within younger participants, we furthermore examined differences between participants in younger adulthood (≤30 years) and midadulthood (31–50 years).

## Materials and methods

The study was conducted according to the principles expressed in the Declaration of Helsinki, obtained ethical and legal approval by the ethics committee at the University of Regensburg (approval number: 20-1786-101), and passed data security inspection by the local data security representative. Reporting follows the STROBE guidelines and checklist for cross-sectional observational studies [37].

### Study design

This observational, online survey-based study was conducted between April 10–27, 2020, during the first COVID-19 lockdown in Southeastern Germany. We used a convenience sample of adults from this region to investigate multiple areas of perceived stress and mental health problems and their clinical relevance during first lockdown, as well as to explore how people feel they have changed in these areas during first lockdown in comparison to before the COVID-19 outbreak ranging from much better to much worse. From April 10, 2020, more than 30,000 confirmed SARS-CoV-2 cases and more than 700 deaths with COVID-19 had been registered in Southeastern Germany, respectively Bavaria [38] (Additional Figure 1). The Bavarian protection measures during first COVID-19 lockdown were the strictest within Germany, and included the prohibition to leave the house without sound reason, and to meet members of another household even outdoors [4] (Fig. 1). The study is part of the ongoing panel study Regensburg Online Study for Mental Health during the COVID-19 Pandemic.

### Setting

The online survey was operated via EvaSys V8.0 (Electric Paper Evaluationssysteme GmbH), and was accessible between April 10–27, 2020. Recruitment was proceeded along the data collection period using different non-probability sampling approaches. First, the survey link was distributed via digital media, social media, the department and university website, the website of the local health office, and the researchers’ social networks using active and passive snowballing. Second, more targeted recruitment was conducted to increase participation of men and non-academics. After accessing the survey via link, participants received detailed information on the study. After giving informed consent by clicking the respective icon, they accessed the questionnaires taking approximately 60 to 90 minutes. As expense allowance, participants were offered a online shopping voucher of five Euros as well as partaking in a lottery to additionally win one of three 100 € vouchers. Participation was voluntary and factually anonymous.

### Participants

Overall, 452 adults from the general population completed the online survey. Inclusion criteria covered a minimal age of 18 years and a place of residence within the German postal code area eight or nine, representing Southeastern Germany and including mainly the Bavarian area. Exclusion criteria entailed a current or past infection with COVID-19 and being currently quarantined at home, since those conditions represented an extraordinary strain and were not representative for the bigger part of the Southern German population at this time of the pandemic. Forty-five participants were excluded due to living outside of the defined region, two participants were excluded due to a current, and three due to a past COVID-19 infection, three participants for being currently home quarantined by official orders, and four participants because information on exclusion criteria was not available. This resulted in a final sample of 396 participants.

### Variables and measures

#### Outcome variables

We focused on the outcome variables worries, tension, joy, and demands as subareas of perceived stress, and the mental health problems depression, anxiety, pathological worry, posttraumatic stress, and health anxiety.

#### State measures during lockdown

State measures during lockdown were assessed using the German versions of standardized questionnaires. The participants’ questionnaire scores during lockdown were categorized as below or above recommended cut-offs from the literature (available for DASS-21 and PTSS-10) to determine their clinical relevance. If no cut-off scores for clinical relevance were available (PSQ-20, PSWQ-PW, and MK-HAI), criterion c thresholds for clinical significance were calculated as approximation. We used the equation $\frac{\left( SD\mathrm{crs}\ast M\mathrm{oss}\right)+\left( SD\mathrm{oss}\ast M\mathrm{crs}\right)}{SD\mathrm{crs}+ SD\mathrm{oss}}$ [39], using means (M) and standard deviations (SD) of our online survey sample (oss) (Table 2), and of clinical reference samples (crs) reported in the literature (Additional Text 1). A questionnaire score above a criterion c threshold calculated with this equation indicates that the respective person is statistically more likely to be from a clinical reference sample than from our general population sample.

Perceived stress was measured using the German modified version of the Perceived-Stress-Questionnaire (PSQ-20) [40], comprising the subscales worries, tension, joy, and demands, all referring to the last four weeks. The measure has been validated in German samples and showed a medium to high consistency (Cronbach’s alpha .80–.86) and split-half-reliability, as well as good convergent and criterion validity [41]. To define criterion c thresholds for the PSQ-20 total score and subscores, we used means and standard deviations from a clinical sample of psychosomatic patients provided in the literature [41] (Additional Text 1). As clinical significance thresholds, 46.37 was calculated for the total score, 44.84 for worries, 45.77 for tension, 44.66 for joy, and 40.02 for demands. Depression and anxiety were measured using the respective subscales of the 21-item Depression-Anxiety-Stress-Scales (DASS21) [42], referring to the last week. The DASS-21 has already been validated for the COVID-19 pandemic in European samples (Poland and Spain), showing good internal consistency [43, 44], split-half reliability, and construct validity [43], also during this particular period. It furthermore has been validated for the German population outside the pandemic, also showing good reliability, as well as construct and structure validity [45]. According to the literature [42], a DASS21 depression subscore ≥10 was defined as indication for a higher probability of a depressive disorder, and an anxiety subscore ≥ six as indication for a higher probability of an anxiety disorder. Pathological worry was measured using the Penn-State-Worry-Questionnaire-Past-Week (PSWQ-PW) [46], assessing worry as typical for generalized anxiety disorder (GAD). The measure was validated in a German sample and showed excellent internal consistency (Cronbach’s alpha 0.84 to 0.93), a lower test-retest-reliability, and a substantial convergent validity [46]. By using the mean and standard deviation from a sample of high-worriers provided in the literature [46] (Additional Text 1), we calculated a criterion c clinical significance threshold of 54.54. Acute and posttraumatic stress symptoms during the last days were measured using the Posttraumatic-Symptom-Scale (PTSS-10) [47]. This questionnaire has been validated in a German sample as well, showing satisfactory to good internal consistency (Cronbach’s alpha 0.79–0.86), a satisfactory test-retest-reliability, and an indication for its validity demonstrated by relations to external criteria [47]. A score ≥24 was defined as suspected PTSD as stated in the literature [47]. Health anxiety was assessed with the German-modified-Health-Anxiety-Inventory (MK-HAI) [48], modified to cover only the last four weeks. The questionnaire has been validated in a German sample and showed a good internal consistency (Cronbach’s alpha 0.93), validity according to high correlations with other measures of health anxiety, but lower correlations with measures of somatic symptoms, illness beliefs, pathological worry, somatization, anxiety, and depression. A criterion c clinical significance threshold of 23.93 was calculated in reference to the mean and standard deviation of a sample with health anxiety indicated by a high Whitely Index [48] (Additional Text 1).

#### Retrospective measures of perceived changes after the COVID-19 outbreak

Adapted versions of the standardized questionnaires were used to measure how people feel they have changed during first COVID-19 lockdown in comparison to before the COVID-19 outbreak. The original questionnaire items of PSQ-20, DASS-21, PSWQ-PW, PTSS-10, and MK-HAI were used, but with a different instruction and rating scale. Participants were now asked to rate their current state during first lockdown compared to their state during the last six months before knowing about SARS-CoV-2. Therefore, a five-point-Likert scale including the following points was provided: –2 *much less than before corona,* –1 *somewhat less,* 0 *equally,* +1 *somewhat more,* +2 *much more than before corona*. For questionnaire items concerning the mental health problems depression (DASS-21), anxiety (DASS-21), pathological worry (PSWQ-PW), PTSD (PTSS-10), and health anxiety (MK-HAI), an additional abstention option (0 *no symptoms now nor before the pandemic*) was given.

Based on these item-specific values (–2 to +2), a participant’s mean change index (–2 *strong decrease* to +2 *strong increase*) was calculated for every outcome variable by averaging the item values of all respective items of the questionnaires. This resulted in individual change indices for PSQ-20 total stress, PSQ-20 worries, PSQ-20 tension, PSQ-20 joy, PSQ-20 demands, DASS-21 anxiety, DASS-21 depression, PSWQ-PW, PTSS-10, and MK-HAI for each participant. Those person-specific change indices were then grouped into three change categories (–2.00 to –0.50 *decrease*, –0.49 to 0.49 *no change*, 0.50 to 2.00 *increase*), and additionally into five more differentiated change categories (–2.00 to –1.50 *strong decrease*, –1.49 to –0.50 *moderate decrease*, –0.49 to 0.49 *no change*, 0.50 to 1.49 *moderate increase*, 1.50 to 2.00 *strong increase*). For PTSS-10, additional change indices were calculated for the questionnaire items on specific (nightmares, jumpiness, and fear of recollection) and non-specific (irritability mood swings, depression, sleep problems, muscular tension, need to withdraw, bad conscience) PTSD symptoms [47] by averaging the change values of the respective questionnaire items into a *specific PTSD symptom-change index* and a *non-specific PTSD symptom-change index*.

#### Further variables and measures

Further variables were (1) sociodemographic information (sex, age range, relationship status, living situation, highest professional qualification, employment status), (2) COVID-19 lockdown related variables (i.e., change in employment status, current work setting, full day childcare responsibility, last face-to-face contact to close relative, fear of losing one’s livelihood [scale 1–5, with five indicating extremely strong fear], fears and worries concerning COVID-19 related to oneself, and related to relatives [each scale 1–7, with seven indicating extremely strong fears]), and (3) health variables (i.e., current mental health treatment, chronic physical diseases). A list of all variables assessed within the comprehensive panel study is available on request.

### Statistical analysis

First, we calculated descriptive statistics of sociodemographic, pandemic-related, and health variables. Second, we calculated means and standard deviations for the outcome variables, concretely for the participants’ questionnaire scores indicating the stress and symptom severity during lockdown, and for the questionnaire specific change indices indicating the direction and degree of on average perceived changes during lockdown in comparison to before the pandemic. Third, two-tailed *t*-tests were conducted to analyze differences in mean change indices between the subareas of perceived stress and between different mental health problems. Alpha was set to .001. We created Kernel density estimation plots [49] to check on the normality assumption for the included variables, and did not observe serious deviations from normality. Forth, we calculated the absolute and relative frequencies of an individually perceived *decrease*, *increase*, or *no change* in outcome variables based on the three change categories, and additionally the absolute and relative frequencies of the more differentiated five change categories. Fifth, we calculated absolute and relative frequencies of participants within a specific change category and a questionnaire score above clinical relevance threshold in relation to all participants and to participants of the respective change category. Sixth, we analyzed significant differences in the proportions of participants within the change category *increase* between the different subscales of perceived stress, and between the different mental health outcome variables. Similarly, we analyzed significant differences in the proportions of participants within the change category *increase* plus additionally showing a questionnaire score above clinical relevance threshold between the different subscales of perceived stress, and between the different mental health outcome variables. Therefore, 99.9% two-sided confidence intervals (CI) for relative frequencies were calculated [50]. Due to a mean decrease in the perceived stress subscale demands within the whole sample, we additionally analyzed significant differences in the proportions of participants within the *decrease*-category between the different subscales of perceived stress. Seventh, we analyzed sex and age specific differences for the main results. Therefore, we created cross tables on the absolute and relative frequencies of men and women (two diverse participants were excluded for these analyses) and of participants with an age >50 years, 31–50 years, and ≤30 years within the change categories *decrease*, *no change*, or *increase*, and within the *increase*-category and a questionnaire score above or below the clinical relevance threshold. We ran χ^2^-tests to compare the respective proportions between sexes and age groups. These analyses were conducted for all outcome variables. The exact significance (two-sided) is reported. Alpha was again set to .001. Furthermore, SPSS cross table z-tests were run to compare the proportion of the total frequencies of the different cells within one row. Different subscripts indicate that these proportions are significantly different. Missing data did not occur for the outcome variables, as questionnaire items were mandatory in the online survey. Missing data among variables for sample characterization are reported in Table 1. Statistical analyses were conducted using SPSS 26 (IBM) and Excel 2016 (Microsoft).

**Table 1:** Sociodemographic, pandemic-associated, and health-related sample characteristics

Characteristics	Total sample (*n*=396)
*Sociodemographic variables*	
Sex, *n* (*%*)	
Female	278 (70.2)
Male	116 (29.3)
Diverse	2 (0.5)
Age categories, *n* (*%*), years	
18–30	138 (34.8)
31–40	101 (25.5)
41–50	54 (13.6)
51–65	79 (19.9)
66–80+	24 (6.1)
Relationship status, *n* (*%*)	
In a relationship	291 (73.5)
Not in a relationship	105 (26.5)
Household size, *n* (*%*)	
Living together with at least one other person	326 (82.3)
Living alone	70 (17.7)
Highest professional qualification, *n* (*%*)^a^	
No professional qualification	51 (12.9)
Completed vocational training	81 (20.5)
Master / technician / comparable	28 (7.1)
University of applied sciences degree	65 (16.5)
University degree	170 (43.0)
Current employment status, *n* (*%*)	
Student	71 (17.9)
Employed	250 (63.1)
Retired or privateer	31 (7.8)
Not employed^b^	30 (7.6)
Other	14 (3.5)
*COVID-19 pandemic associated variables*	
Change in employment status due to lockdown, *n* (*%*)	
No change	235 (59.3)
Furlough	21 (5.3)
Reduction of occupational activity	83 (21.0)
Discontinuation of occupational activity	27 (6.8)
Elevation of occupational activity	30 (7.6)
Work setting during lockdown, *n* (*%*)^c^	
Home-office (without childcare)	137 (34.6)
Home-office (additional childcare)	25 (6.3)
At-place (low contact)	50 (12.6)
At-place (high contact)	35 (8.8)
At-place, medical area (no COVID-19 patients)	35 (8.8)
At-place, medical area (COVID-19 patients)	20 (5.1)
(Currently) not working^d^	94 (23.7)
Full day childcare responsibility during lockdown, *n* (*%*)	
Yes	65 (16.4)
No	331 (83.6)
Live-contact with close person within last week, *n* (*%*)	
Yes	249 (62.9)
No	147 (27.1)
Fear of losing one’s livelihood – score (1-5), *M* (*SD*)	2.09 (1.09)
Fear and worries COVID-19 self – score (1-7)^e^, *M* (*SD*)	3.06 (1.29)
Fear and worries COVID-19 relatives – score (1-7)^f^, *M* (*SD*)	4.59 (1.54)
*Health variables*	
Current mental health treatment^g^, *n* (*%*)^h^	
Yes	55 (14.0)
No	338 (85.4)
Chronic physical diseases^i^, *n* (*%*)	
Yes	118 (29.8)
No	278 (70.2)

*COVID-19* coronavirus disease 2019

^a^ One missing value, *n*=395

^b^ Includes being housewife/husband, on permanent sick leave/unable to work, permanently jobless, or welfare recipient

^c^ Percentages do not add to 100% due to rounding

^d^ Includes not having an occupation or not being a student, as well as sick-leave and lockdown-associated furlough

^e^ An average score over four rating items including body checking on COVID-19 associated symptoms, disconcertment in case of sensing COVID-19 associated symptoms, fear of own illness with COVID-19, and fear of own death from COVID-19 (item values 1–7) was calculated

^f^ An average score over three rating items including fear of illness of a relative with COVID-19, fear of a relative’s death from COVID-19, and fear of not being able to care for relatives being ill with COVID-19 (item values 1–7) was calculated

^g^ Includes current psychiatric, psychotherapeutic, or psychopharmacological treatment

^h^ Three missing values, *n*=393

^i^ Includes cardiovascular diseases, respiratory diseases, diabetes, liver or kidney diseases, carcinosis, or diseases of immunity

## Results

### Sample characteristics

The analysis sample comprised 396 adult participants, 278 female (70.2%), 116 male (29.3%), and two diverse (0.5%). Table 1 displays sociodemographic, pandemic-associated, and health variables. Table 2 presents descriptive statistics on the outcome variables for the whole sample.

**Table 2:** Stress and mental health during first COVID-19 lockdown and changes compared to before the pandemic

Variables	Total sample^a^		Change category subsamples^d^								
			Perceived decrease			Perceived no change			Perceived increase		
	Score^b^	Clin. rel.^c^		Score^b^	Clin. rel.^c^		Score^b^	Clin. rel.^c^		Score^b^	Clin. rel.^c^
	*M* (*SD*)	*n* (*%*)	*n* (*%*)^e^	*M* (*SD*)	*n* (*%*)	*n* (*%*)^e^	*M* (*SD*)	*n* (*%*)	*n* (*%*)^e^	*M* (*SD*)	*n* (*%*)
Perceived stress											
Total	39.66 (21.44)	143 (36.1)	60 (15.2)	24.42 (17.25)	6 (1.5)	233 (58.8)	34.34 (17.42)	57 (14.4)	103 (26.0)	60.57 (16.62)	80 (20.2)
*Worries*	36.75 (25.78)	140 (35.4)	32 (8.1)	25.42 (23.0)	5 (1.3)	224 (56.6)	26.37 (19.23)	39 (9.8)	140 (35.4)	55.95 (24.54)	96 (24.2)
*Tension*	40.76 (27.02)	171 (43.2)	66 (16.7)	21.62 (20.62)	8 (2.0)	203 (51.3)	32.38 (21.51)	59 (14.9)	127 (32.1)	64.09 (21.76)	104 (26.3)
*Joy*	52.61 (23.89)	141 (35.6)	151 (38.1)	37.79 (20.45)	94 (23.7)	205 (51.8)	60.59 (21.32)	43 (10.9)	40 (10.1)	67.67 (19.42)	4 (1.0)
*Demands*	33.74 (25.31)	132 (33.3)	155 (39.1)	18.67 (17.64)	16 (4.0)	169 (42.7)	34.75 (20.59)	57 (14.4)	72 (18.2)	63.80 (21.69)	59 (14.9)
Mental health problems											
Depression	4.68 (4.70)	55 (13.9)	14 (3.5)	5.57 (5.77)	2 (0.5)	279 (70.5)	2.94 (3.20)	12 (3.0)	103 (26.0)	9.29 (4.84)	41 (10.4)
PTSD	19.57 (12.88)	142 (35.9)	12 (3.0)	19.25 (15.69)	3 (0.8)	283 (71.5)	15.59 (10.59)	66 (16.7)	101 (25.5)	30.75 (11.87)	73 (18.4)
Anxiety	2.16 (2.90)	42 (10.6)	9 (2.3)	3.22 (4.52)	2 (0.5)	365 (92.2)	1.76 (2.28)	22 (5.6)	22 (5.6)	8.36 (4.08)	18 (4.5)
Path. Worry	37.83 (18.73)	83 (21.0)	11 (2.8)	36.45 (16.48)	2 (0.5)	346 (87.4)	35.51 (17.81)	54 (13.6)	39 (9.8)	58.82 (13.88)	27 (6.8)
Health Anxiety	17.19 (11.07)	113 (28.5)	3 (0.8)	23.33 (6.66)	1 (0.3)	364 (91.9)	15.86 (10.24)	86 (21.7)	29 (7.3)	33.28 (8.39)	26 (6.6)

*COVID-19* coronavirus disease 2019, *PTSD* posttraumatic stress disorder, *Path* pathological, *Clin. rel* clinically relevant

^a^ *N*=396 participants (100.0%)

^b^ Questionnaire scores for PSQ-20 (Perceived-Stress-Questionnaire) total, and the subscales worries, tension, joy, and demands (each range 0–100), DASS21 (Depression-Anxiety-Stress-Scales) subscales depression and anxiety (each range 0–21), PTSS-10 (Posttraumatic-Symptom-Scale; range 0–60), PSWQ-PW (Penn-State-Worry-Questionnaire-Past-Week; range 0–90), and MK-HAI (German-modified-Health-Anxiety-Inventory; range 0–56), all assessing the participants’ state during lockdown

^c^ Number and percentage (within the total sample) of participants with clinically relevant questionnaire scores during lockdown according to cut-offs stated by the questionnaire authors (≥10 for DASS21 depression, ≥24 for PTSS-10, and ≥6 for DASS21 anxiety), or according to criterion c thresholds for clinical significance calculated with reference to clinical samples from the literature (≥46.37 for PSQ-20 total, ≥44.84 for PSQ-20 worries, ≥45.77 for PSQ-20 tension, ≥44.66 for PSQ-20 joy, ≥40.02 for PSQ-20 demands, ≥54.54 for PSWQ-PW, and ≥23.93 for MK-HAI)

^d^ Adapted versions of the stated questionnaires were used to measure perceived changes during first lockdown in comparison to before the COVID-19 pandemic on item level (–2 *much less than before corona*; +2 *much more than before corona*). Change indices (–2 *strong* decrease; +2 *strong* increase) were calculated for each participant and each outcome variable by averaging the change values for the respective questionnaire items, and were grouped into three change categories (–2.00 to –0.50 *decrease*; –0.49 to +0.49 *no change*; +0.50 to +2.00 *increase*)

^e^ Absolute and relative frequencies of participants with a perceived decrease, no change, or increase in perceived stress and mental health problems during first lockdown in comparison to before the COVID-19 pandemic

### Main results

#### Perceived changes in stress and clinical relevance during lockdown

The mean change indices (range –2 *strong decrease* to +2 *strong increase*) for individually perceived changes in total perceived stress (*M*=0.14, *SD*=0.64), and in the subscales worries (*M*=0.34, *SD*=0.67), tension (*M*=0.18, *SD*=0.83), and lack of joy (joy inverted) (*M*=0.32, *SD*=0.72) showed that on average, participants experienced an increase during lockdown in comparison to before the pandemic, while demands were on average experienced to be decreased (*M*=–0.29, *SD*=0.94). *t*-Tests showed a significant difference between the change indices for demands and all other subscales (*ps*<.001). Furthermore, the on average perceived increases in worries and lack of joy were both higher than in tension (*ps*<.001), with no significant difference between them (*p*=.404).

**Fig. 2:**
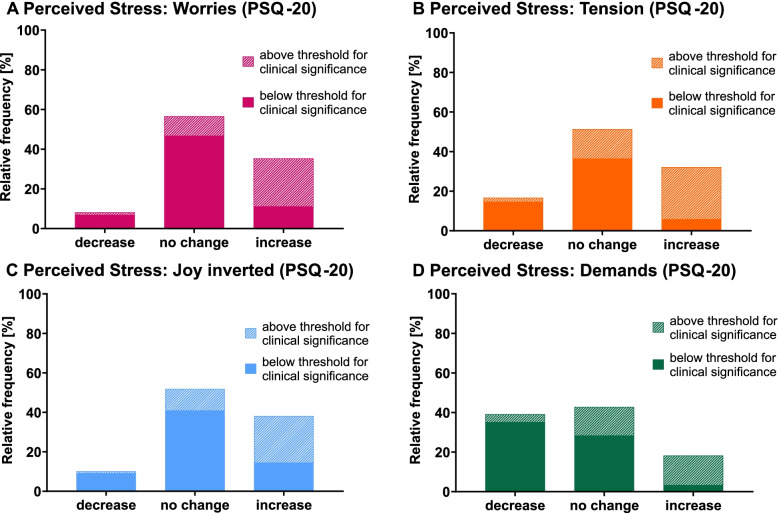
Perceived changes in stress and clinical relevance during first COVID-19 lockdown. *N*=396 adult participants. Lengths of the single bars (comprising shaded and non-shaded parts) represent the relative frequencies of individually perceived decreases, no changes, and increases in subareas of perceived stress during first lockdown in comparison to before the COVID-19 pandemic. Lengths of the shaded parts within the single bars represent the respective percentage of participants whose level of perceived stress during lockdown reached clinical relevance thresholds. The non-shaded parts of the single bars represent the respective percentage of participants whose level of perceived stress during lockdown was below clinical relevance thresholds. To determine frequencies of perceived decreases, no changes, and increases in subscales of perceived stress, a modified version of the PSQ-20 was used to assess how people feel their stress has changed during lockdown in comparison to before the pandemic on item level (change values; –2 *much less than before corona*; +2 *much more than before corona*). Change indices (–2 *strong* decrease; +2 *strong* increase) were calculated for each participant and each outcome variable by averaging the change values for the respective subscales’ questionnaire items, and were grouped into three change categories (–2.00 to –0.50 *decrease*; –0.49 to +0.49 *no change*; +0.50 to +2.00 *increase*). The original version of PSQ-20 was used to measure the intensity of worries, tension, joy, and demands during first COVID-19 lockdown. To determine the clinical relevance of these subscales of perceived stress during lockdown, the participants’ questionnaire scores were classified as above or below clinical significance thresholds calculated in reference to a clinical sample from the literature (≥46.37 for PSQ-20 total, ≥44.84 for PSQ-20 worries, ≥45.77 for PSQ-20 tension, ≥44.66 for PSQ-20 joy, and ≥40.02 for PSQ-20 demands). The subscale joy was inverted for presentation in this figure, indicating lack of joy.

Regarding frequencies (Fig. 2, Table 2), decreases (change indices between –2.00 to –0.50) in total perceived stress were reported by 15.2% of all participants, decreases in worries by 8.1% (99.9% CI, 3.6%-12.6%), in tension by 16.7% (99.9% CI, 10.5%-22.9%), in lack of joy by 10.1% (99.9% CI, 5.1%-15.1%), and in demands by 39.1% (99.9% CI, 31.0%-47.2%). The relative frequency of participants experiencing a decrease in demands was larger than for all other subscales (*ps*<.001), with no significant differences between those. An increase (change indices between +0.50 to +2.00) in total perceived stress was reported by 26.0% of all participants, an increase in worries by 35.4% (99.9% CI, 27.5%-43.3%), in tension by 32.1% (99.9% CI, 24.4%-39.8%), in lack of joy by 38.1% (99.9% CI, 30.1%-46.1%), and in demands by 18.2% (99.9% CI, 11.8%-24.6%). The relative frequencies of perceived increases in worries, tension, and lack of joy, as well as in tension and demands did not differ significantly, but relative frequencies of perceived increases in worries and lack of joy were higher than in demands (*p*s<.001).

Perceived increases going along with a clinically relevant stress level during lockdown (criterion c thresholds see Materials and methods) were found for total perceived stress in 20.2% of all participants, for worries in 24.2% (99.9% CI, 17.1%-31.3%), for tension in 26.3% (99.9% CI, 19.0%-33.6%), for lack of joy in 23.7% (99.9% CI,16.7%-30.7%), and for demands in 14.9% (99.9% CI, 9.0%-20.8%) (Fig. 2, Table 2). The proportions did not differ significantly. Table 2 and Additional Table 1 report further results.

#### Perceived changes in mental health problems and clinical relevance during lockdown

Mean change indices (range –2 *strong decrease* to +2 *strong increase*) for individually perceived changes showed that on average, participants experienced an increase in mental health problems during lockdown in comparison to before the pandemic. The on average perceived increases in depression (*M*=0.27, *SD*=0.56) and posttraumatic stress (*M*=0.23, *SD*=0.44) were both higher than in health anxiety (*M*=0.11, *SD*=0.24), pathological worry (*M*=0.10, *SD*=0.34), and anxiety (*M*=0.06, *SD*=0.34) (*ps*<.001), with no significant difference between increases in depression and posttraumatic stress (*p*=.139). Between health anxiety and pathological worry (*p=*.626), pathological worry and anxiety (*p*=.029), and health anxiety and anxiety (*p*=.003), we found no significant differences in the on average perceived increases.

**Fig. 3:**
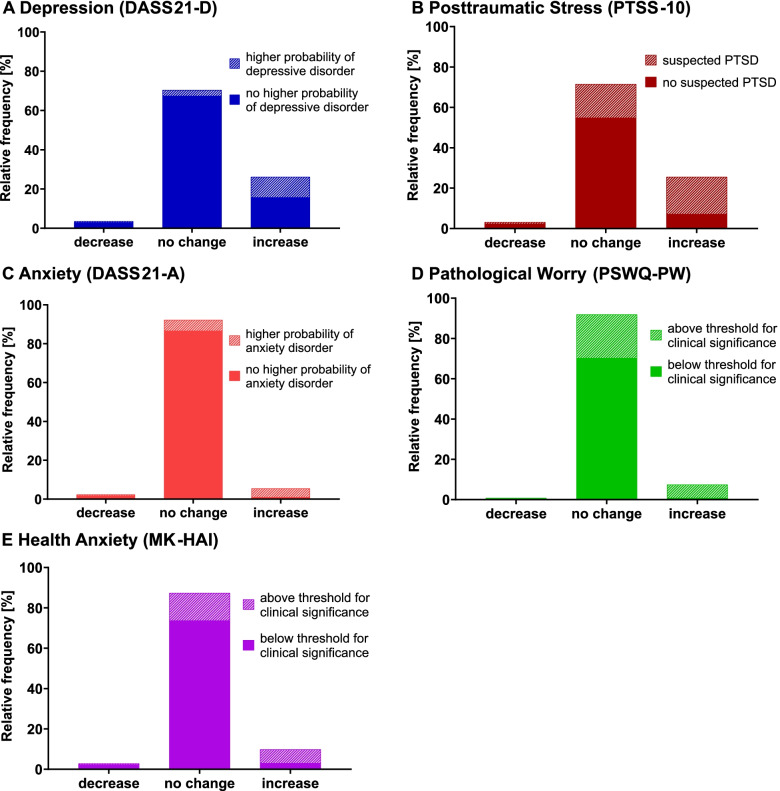
Perceived changes in mental health problems and clinical relevance during first COVID-19 lockdown. N=396 adult participants. Lengths of the single bars (comprising shaded and non-shaded parts) represent the relative frequencies of individually perceived decreases, no changes, and increases in mental health problems during first lockdown in comparison to before the COVID-19 pandemic. Lengths of the shaded parts within the single bars represent the respective percentage of participants whose level of symptom severity during lockdown reached clinical relevance thresholds. The non-shaded parts of the single bars represent the respective percentage of participants whose symptom level was below clinical relevance thresholds. To determine frequencies of perceived decreases, no changes, and increases in mental health problems, modified versions of the questionnaires DASS21 depression, PTSS10, DASS21 anxiety, PSWQ-PW, and MK-HAI were used to assess how people feel their mental health has changed during lockdown in comparison to before the pandemic on item level (change values; –2 much less than before corona; +2 much more than before corona). Change indices (–2 strong decrease; +2 strong increase) were calculated for each participant and each outcome variable by averaging the change values for the respective questionnaire items, and were grouped into three change categories (–2.00 to –0.50 decrease; –0.49 to +0.49 no change; +0.50 to +2.00 increase). The severity of symptoms during lockdown was measured using the original questionnaires (DASS21 depression, PTSS-10, DASS21 anxiety, PSWQ-PW, and MK-HAI). To determine the clinical relevance of the symptom severity, the participants’ questionnaire scores were classified as above or below cut-offs stated by the questionnaire authors (≥10 for DASS21 depression, ≥24 for PTSS-10, and ≥6 for DASS21 anxiety), or as above or below clinical significance thresholds calculated in reference to clinical samples from the literature (≥54.54 for PSWQ-PW, and ≥23.93 for MK-HAI)

Regarding frequencies (Fig. 3, Table 2), an increase (change indices between +0.50 to +2.00) in depression was reported by 26.0% of all participants (99.9 % CI, 18.7%-33.3%), an increase in posttraumatic stress by 25.5% (99.9 % CI, 18.3%-32.7%), in pathological worry by 9.8% (99.9 % CI, 4.9%-14.7%), in health anxiety by 7.3% (99.9 % CI, 3.0%-11.6%), and in anxiety by 5.6 % (99.9 % CI, 1.8%-9.4%). Increases in depression and posttraumatic stress were both reported more frequently than in pathological worry, health anxiety, and anxiety (*p*s<.001), while all other frequencies did not differ significantly. Decreases (change indices between –2.00 to –0.50) in mental health problems were reported by 3.5% of all participants for depression, by 3.0% for posttraumatic stress, by 2.3% for anxiety, by 2.8% for pathological worry, and by 0.8% for health anxiety.

Perceived increases in mental health problems going along with a clinically relevant symptom severity during lockdown (cut-offs and criterion c thresholds see Materials and methods) were found for posttraumatic stress in 18.4% of all participants (99.9 % CI, 12.0%-24.8%), for depression in 10.4 % (99.9% CI, 5.4%-15.4%), for pathological worry in 6.8% (99.9% CI, 2.6%-11.0%), for health anxiety in 6.6% (99.9% CI, 2.5%-10.7%), and for anxiety in 4.5% (99.9% CI, 1.1%-7.9%) (Fig. 3, Table 2). The proportion was higher for posttraumatic stress than for pathological worry, health anxiety, and anxiety (*p*s<.001), while all other frequencies did not differ significantly. Table 2 and Additional Table 2 display further results.

**Fig. 4:**
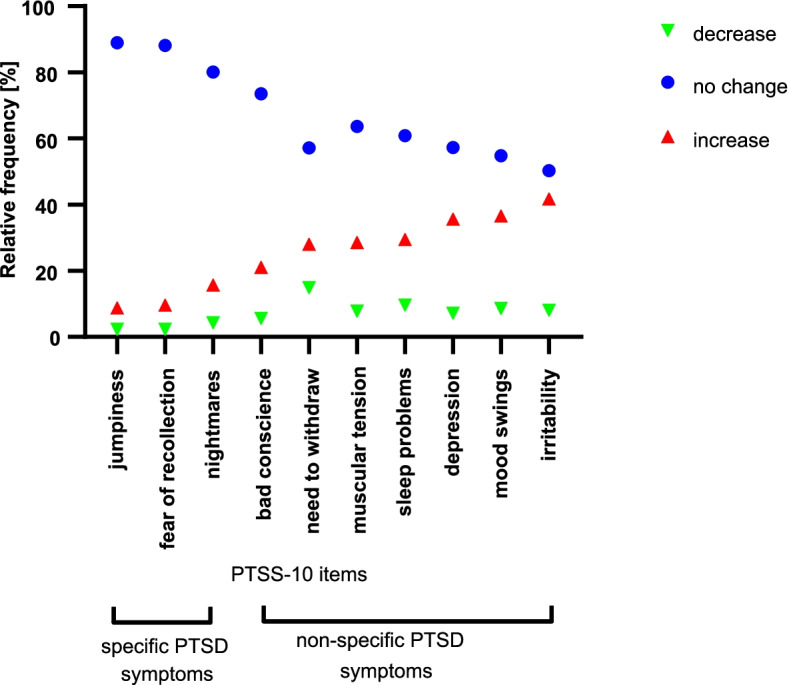
Perceived changes in specific and non-specific PTSD symptoms during compared to before the COVID-19 pandemic. *N*=396 adult participants. The symbols indicate the relative frequencies of participants reporting a decrease, no change, or increase in different posttraumatic stress disorder (PTSD) symptoms. To determine frequencies of individually perceived decreases, no changes, and increases, a modified version of the 10-item Posttraumatic Symptom Scale (PTSS-10) was used to assess how people feel symptoms have changed during lockdown in comparison to before the pandemic on item level. For each participant, the change values for the single questionnaire items (–2 *much less than before* corona; +2 *much than before* corona) were grouped into three change categories (–2.00 to –0.50 *decrease*, –0.49 to +0.49 *no change,* +0.50 to +2.00 *increase*). Jumpiness, fear of recollection, and nightmares were classified as specific symptoms of PTSD, all other PTSS-10 symptoms as non-specific PTSD symptoms [47]

Regarding different PTSD symptoms, the relative frequency of a perceived increase in non-specific PTSD symptoms (irritability [41.7%]; mood swings [36.6%]; depression [35.6%]; sleep problems [29.5%]; muscular tension [28.5%]; need to withdraw [28.0%]; bad conscience [21.0%]; non-specific PTSD symptoms-change index [27.8%; 99.9% CI, 20.4%-35.2%]) was higher than in specific PTSD symptoms (nightmares [15.7%], fear of recollection [9.6%], jumpiness [8.8%]; specific PTSD symptoms-change index [10.6%; 99.9% CI, 5.5%-15.7%]) (*p*<.001). Figure 4 displays individually perceived changes in the single specific and non-specific PTSD symptoms.

### Additional analyses

#### Sex specific differences

We did not find significant associations between the participants’ sex (male; female) and changes in perceived stress and mental health problems from before to during lockdown (decreases; no changes; increases), except for demands as subarea of perceived stress, χ^2^=14.45, *p*<.001. A significantly higher percentage of women than men reported increases in external demands from before to during lockdown, and a significantly higher percentage of men than women reported no change (Additional Table 3). Concerning an increase going along with a clinically relevant stress or symptom level during lockdown, no significant associations with the participants’ sex were found (Additional Table 4).

#### Age specific differences

For participants’ age (≤30; 31–50; >50 years), we did not find significant associations with changes in perceived stress and mental health problems during lockdown (decreases; no changes; increases), except for demands as a subarea of perceived stress, χ^2^=31.06, *p*<.001. Perceived increases in external demands were significantly more frequent in participants aged 31–50 years than in participants aged ≤30 and >50 years, decreases were significantly more frequent in participants aged ≤30 years than in participants aged 31–50 and >50 years, and no changes were significantly more frequent in participants aged >50 years than 31–50 and ≤30 years (Additional Table 5). Concerning increases in stress or mental health problems going along with a clinically relevant stress or symptom level during lockdown, we did not find significant associations with the participants’ age group (Additional Table 6).

## Discussion

This study explored how people feel their stress and mental health has changed during the first COVID-19 lockdown in comparison to before the pandemic using a convenience sample of adults from Southeastern Germany, as the region where SARS-CoV-19 was first documented in Germany, and where the strictest lockdown measures within Germany were implemented. In comparison to the majority of previous studies on adults from the general population, we observed individually perceived changes instead of comparing prevalence rates or sample means before and during the pandemic. Furthermore, we assessed multiple outcome measures to be able to compare the extent of individually perceived changes between different subareas of perceived stress and mental health problems.

### Summary and interpretation of results

#### Dissociation of changes in subareas of perceived stress

As first key finding, we found differences in perceived changes between subareas of perceived stress. While worries, tension, and lack of joy were on average experienced to be increased, demands were on average experienced to be decreased. Regarding frequencies, about one third of all participants reported an increase in worries, tension, and lack of joy, and one sixth a decrease. For demands, less than 20% reported an increase, but about 40% a decrease. While worries, tension, and lack of joy represent internal stress reactions, the demands subscale of PSQ-20 reflects perceived external stressors like lack of time, pressure, and overload [51]. Previous studies found a general increase in psychosocial stress during the early phase of the COVID-19 pandemic among the general population of Germany [19, 20] and other countries [52], while another German study [27] and two worldwide meta-analyses [17, 18] did not detect a significant change in general distress. Since changes in different subareas of perceived stress have not yet been compared, this study is the first to show a dissociation of individually perceived changes of subareas, possibly explicable by characteristics of the lockdown situation. The spreading of SARS-CoV-2, a reduction of social contact, home office, or reduced occupational activity might reduce external demands such as time pressure on the one hand, but on the other hand induce worries, tension, and a lack of joy. Interestingly, perceived decreases in external demands were more frequent in younger (18–30 years) than in older adults (≥31 years). Increases in external demands were most frequently experienced by participants in midadulthood (31–50 years), as well as by women. There might be associations with gender roles as well as with the living conditions of individuals of different sexes and age groups during lockdown, which could be examined in future studies investigating on mechanisms behind differential reactions to the pandemic lockdown.

#### Varying extent of increases in different mental health problems

As a second key finding, around one quarter of all participants reported an increase in depressive and PTSD symptoms from before to during the pandemic, while only around five to ten percent reported an increase in anxiety, pathological worry, and health anxiety symptoms. Previous research on mental health during the early phase of the COVID-19 pandemic found increases in depression [19–23], anxiety [19–24], and health anxiety [25, 26] among the general population of Germany, as well as increases in anxiety and depression [14–18], and health anxiety [34] among the general population also of other countries. Other German studies found no significant changes [27], or even a decrease [30] in general mental health problems. Increases in PTSD were found in countries outside of Germany [14], while results on changes from before to during the pandemic within adults from the German general population have not been published yet. Also, specific data on changes in pathological worry in the general population have not yet been reported for the COVID-19 pandemic. Moreover, as previous studies mainly compared sample means or prevalence rates from before and during the COVID-19 pandemic, there are only few studies on changes at the level of the individual. Those reports found subgroups of participants with deteriorations but also with improvements in general mental health during the early phase of the COVID-19 pandemic [32, 33, 35], but did not discriminate between different mental health syndromes. Therefore, a dissociation of individually perceived increases with significantly larger and more frequently reported increases in depressive and posttraumatic stress symptoms than in anxiety, pathological worry, and health anxiety symptoms represents a novel finding of our study and demands for discussion.

Although our individual-level approach limits the comparability to previous studies, the considerable amount of participants reporting an increase in depressive symptoms seems to confirm previous results of a heightened prevalence of depression during in comparison to before the early phase of the COVID-19 pandemic [e.g., 14.3% vs. 5.6% [19], 14.3% vs 7.6% [20], or 31.4% vs. 8.1% [22] among the German general population]. The higher and more frequent perception of increases in depression than in anxiety in our sample is in accordance with a meta-analysis on longitudinal studies from Europe and North America showing a significantly larger increase for depressive than for anxiety symptoms from before to during the pandemic [18], though no individual changes but prevalence rates were analysed in this work.

To further interpret our finding of lower increases in anxiety, pathological worry, and health anxiety, the measurement instruments and constructs measured need to be considered as well: First, we assessed health anxiety in general, not in relation to COVID-19 in particular, perhaps explaining the lower extent of perceived increases in this symptom area within the participants of our sample. Cyberchondria and hypochondrial safety behavior specifically related to COVID-19 [25, 26] might be more suitable concepts for future studies, however, validated questionnaires on these constructs are yet to be established. Second, the DASS21 anxiety subscale used to assess anxiety within our study measures symptoms of acute fear and physical hyperarousal (e.g., tachycardia, xerostomia, or shakiness) as associated to panic disorder [53], and the PSWQ-PW [46] used to assess pathological worrying measures the excessiveness, duration, and uncontrollability of worry as cognitive subcomponent of generalized anxiety disorder (GAD). Both questionnaires do not assess physical symptoms of persistent arousal and tension (e.g., irritability or muscular tension) as associated to stress and as representing a further subcomponent of GAD [54, 55]. Previous studies from Germany on increases in anxiety during the COVID-19 pandemic predominantly focused on generalized anxiety using the GAD-2 [20, 21, 23], or GAD-7 [19, 24]. Both questionnaires include items on cognitive and physical symptoms of GAD [56, 57]. Those studies found more additional cases and a higher absolute prevalence of GAD during in comparison to before the pandemic [16.8% vs 6.0% [19], 19.7% vs 9.0% [20], eight times higher [24]] than another German study found for panic disorder and other anxiety disorder measured with the PHQ-D [5.7% vs 2.0% and 7.4% vs 2.2% [22]]. Furthermore, a meta-analysis on studies from China and other countries (without Germany) showed an average prevalence of anxiety during the COVID-19 pandemic of 23.4% in studies using the DASS21, which was lower than the average prevalence of 40.7% in studies using the GAD-7, and lower than the 44.5% found in studies using other tools for anxiety measurement (SAS, HADS, Likert-scale) [58]. We therefore hypothesize that during the COVID-19 pandemic, symptoms of acute fear and panic, but also pathological worrying as cognitive subcomponent of GAD, might be less affected than physical symptoms of general anxiety.

This hypothesis finds support from our more differentiated analysis of increases in PTSD symptoms within our sample, in which we separated PTSD symptoms assessed with the PTSS-10 into general stress symptoms and symptoms specifically related to experienced traumata [47]. Interestingly, our participants more frequently reported increases in non-specific than in specific PTSD symptoms. These non-specific PTSD symptoms (e.g., irritability, sleep problems; muscular tension, or need to withdraw) are also associated with depressive and other affective disorders, adjustment disorders, and GAD [59]. Therefore, our PTSS-10 results should not be interpreted with regards to PTSD in particular, but as a more general stress reaction towards the pandemic and lockdown. Our result for tension as subarea of perceived stress (PSQ-20), which was already discussed above, points in this direction as well. Tension represents a specific, physical aspect of stress and general anxiety and was frequently increased in our sample.

This leads to the question, why worries as further subscale of perceived stress (PSQ-20) were more frequently increased than pathological worrying (PSWQ-PW). To resolve this contradiction, we can argue that pathological worrying represents a specific, pathological meta-cognitive process characterizing mental processes of individuals suffering from a mental disorder like generalized anxiety disorder (GAD). Worries as a subaspect of perceived stress, in contrast, are a broader construct and such worries could also be found in healthy individuals. This differences between constructs could explain the larger increase in stress associated worry than in pathological worry.

Altogether, our findings imply substantial individually perceived increases in symptoms of depression and general stress during first COVID-19 lockdown in our convenience sample of adults from Southeastern Germany, and that the extent of these perceived increases was larger than increases in specific PTSD symptoms, symptoms of acute fear and panic, pathological worry as cognitive component of GAD, and general hypochondria. Since a decreased availability of potential reinforcers (e.g., social, physical, or other pleasant events) is an well-established etiological factor for depressive symptoms [60, 61], an increase in this specific symptom area was to be expected during a lockdown with stay-at-home orders and social contact restrictions. Supporting this notion, associations between depression and reduced physical activity [62], and social contact [63] during the COVID-19 pandemic have previously been found. Indeed, reduced social contact during the pandemic was also associated to generalized anxiety [63], supporting the hypothesis of a high impact of pandemic conditions not only on depressive but also on general stress symptoms represented within the GAD criteria. Furthermore, the relatively low increase in specific PTSD symptoms is not surprising, considering PTSD only develops following exposure to an extremely threatening or horrific event or series of events [64]. Although the COVID-19 pandemic lockdown represented an extreme stressor for certain individuals, it might only fulfill the criteria for a traumatic event in seldom cases, e.g., suffering from a life-threatening illness with COVID-19, or experiencing the severe illness or death of a relative with COVID-19. Since PTSD symptoms may appear several weeks after trauma exposure, follow-up assessments after first lockdown might furthermore be necessary to detect all cases of PTSD following first lockdown.

#### Clinical severity of increased stress and mental health problems

As third key finding, perceived increases in mental health symptoms going along with a symptom severity of clinical relevance during lockdown were most frequently found for depression with one out of ten (10.4%), and for PTSD with nearly two out of ten participants (18.4%). Again, it is to consider that mainly non-specific PTSD symptoms associated to a general stress response were experienced to be increased within our sample. Perceived increases in anxiety, pathological worry, and health anxiety going along with a symptom severity of clinical relevance during lockdown were found in 4.5–6.8% of all participants, and in 14.9–26.3% for subareas of perceived stress. In comparison to the previous studies mentioned above, we particularly report on clinically relevant stress and mental health problems in participants reporting a stress or symptom increase during lockdown.

Subject to future confirmation in samples representative for the general population, our results suggest an additional need for mental health care during the COVID-19 pandemic, and that frequently affected mental health problems (like depressive and general stress symptoms within our sample) should be specifically targeted. Within a commentary on mental health strategies to combat the psychological impact of the COVID-19 pandemic [65], the identification of high-risk groups, an improved screening of mental disorders as comorbidities, cognitive behavioral therapy and mindfulness-based therapy to target mental health issues, as well as the dissemination of health-related information for the public (e.g., on how to emotionally cope with fear of the virus) were suggested. Since we already mentioned reduced social contact as risk factor for increases in depression and other mental health problems, prevention strategies specifically targeting social isolation should be considered as well [66]. As examples for interventions during the COVID-19 pandemic, a systematic review showed that psychological therapies like mindfulness, lessons on friendship, robotic pets, and social facilitation software could be effective in reducing loneliness, and that mindfulness therapy, visual art discussions, Tai Chi Qigong meditation, and a cognitive enhancement program can be effective in improving social support [67]. To prevent a further spreading of the virus, video consultations and other telemental health services through e-mail, telephone, or smartphone apps should be discussed as promising options [65, 68, 69] for the implementation of intervention and prevention strategies.

#### Decreases in stress and mental health problems

Lastly, 39.1% of all participants reported individually perceived decreases in external demands, 8.1–16.7% decreases in worries, tension, and lack of joy, and 0.8–3.5% decreases in mental health problems during first COVID-19 lockdown in comparison to before the pandemic. While no previous results exist on changes in subareas of perceived stress, a previous study on the German general population has also found an individual decrease in overall mental health problems in 2.5% of their participants [35]. In studies on the general population of other countries, overall mental health problems were reported to be a little better in 3.8% and a lot better in 0.7% of Australian participants [34], and were reported to be decreased in 3.6% of an UK sample [35]. One might speculate that decreases in mental health problem may be due to decreased external demands during lockdown. As one potential pathway, decreases in external demands might facilitate more time for (virtual) social contact, having an positive influence on mental health. As an alternative explanation, decreases in demands and reductions in social contact during lockdown might have brought some kind of relief to some participants, e.g., participants already suffering from mental health problems like social phobia or depression prior to the pandemic, thereby resulting in decreased mental health problems during lockdown in these participants. Previous studies specifically examining changes in participants with preexisting clinically significant mental health problems found mixed results. A German study found increased sample means for generalized anxiety and depression from before to during the pandemic [70]. In contrast, a meta-analysis on studies from different countries reported no significant change in sample means for mental health problems among participants with pre-existing mental health conditions, speculating about a naturally occurring recovery or a more structured routine and less external stressors due to stay-at-home orders as a potential explanations [18]. As examples for results on individual-level changes, a study from the Netherlands found a worsening of preexisting moderate to high symptoms of anxiety and depression in 24.2%, no change in 54.3%, an improvement in 5.4%, and a remission in 16.1% [32]. Altogether, decreases in subareas of perceived stress (like external demands) and in mental health problems, as well as their relations and potentially associated conditions and mechanisms demand for further investigation.

### Strengths and limitations

As one main strength of our study, we analyzed changes at the level of the individual, and thereby expected to capture more valid results which are likely to be overlooked in comparisons of sample means or prevalence rates from before and during the pandemic. As main limitation, we conducted retrospective assessments of how people feel they have changed from before to after the COVID-19 outbreak, implying the risk of recall bias. For patients’ recall of their health state, in particular, studies have shown inconsistencies between initial and recalled assessments of symptoms [71]. For individuals with a history of depression in particular, a more pronounced overestimation of previously experienced negative emotions was found compared to participants without a history of depression [72]. To reduce recall bias, we did not request the participants to report on how they believe their mental condition has been before the pandemic (e.g., by rating the intensity of symptoms experienced before the pandemic on a Likert scale from *not at all* to *extremely*), as conducted in some retrospective studies on mental health during the pandemic [20, 26]. Instead, we asked them to report on individually perceived *changes* during first lockdown in comparison to the last six months before knowing about SARS-COV-2 (by rating symptoms on a Likert scale from *much less than before corona* to *much more than before corona*), similar to some other retrospective studies on the COVID-19 pandemic [34, 35]. We applied this rating format for every questionnaire item, and expected that the feeling of being worse or better is easier to report than recalling the exact symptom severity before the pandemic outbreak. The eligibility of such health transition items is supported by a study comparing a single item retrospective evaluation of subjective health change (*much worse* to *much better*) with prospective assessments by means of a health survey questionnaire, finding a linear association and suggesting that both measurements are sensitive to true changes [73]. As further risk for bias, specifically asking about perceived changes after the COVID-19 outbreak might entail confirmation bias. Participants might expect the pandemic to negatively impact mental health, and thereby might have focused on perceptions confirming this presupposition during answering change-related questions, possibly leading to an overestimation of increases in stress and mental health. After all, it remains uncertain if our findings may be considered as reliable indicators of true changes, but they certainly inform us about how participants feel they have changed after the COVID-19 outbreak.

As further strength of our study, we measured individually perceived changes in multiple outcome measures for the first time and were thereby able to compare the extent and frequency of individually perceived increases and decreases between different areas of stress and mental health. In doing so, our findings can form future research hypotheses, best examined in longitudinal studies as a next step. As further disadvantage of directly assessing perceived changes from worse to better, we could only assess the clinical relevance of stress and mental health problems for the lockdown, but not for the pre-pandemic time period. Because not all questionnaires stated validated cut-offs for clinical relevance, we furthermore calculated clinical significance thresholds in reference to clinical samples from the literature, which may only be interpreted as an approximate. While validated self-report measures were used, these do also not allow secured mental disorder diagnosis. However, the urgent situation and the social contact prohibitions impaired the realization of standardized interviews. For the same reasons, we did not assess biological markers, which could be an interesting extension for psychometric assessments of mental health during the COVID-19 pandemic.

As further limitation, the generalizability of our results must be interpreted with caution given the sample size, the distribution of sociodemographic characteristics, and the non-probability sampling method, all due to the rapid collection of data during the early phase of the COVID-19. Although we tried for a balanced sampling, the sample was not representative for the general population of Southeastern Germany. There was an overrepresentation of younger participants and women in comparison to the distribution in the general population of Germany aged 18 years or older as published by the German Federal Statistical Office [74], the latter of which is a well-known problem in scientific studies [75]. To control for potential sex and age specific differences, we conducted additional subgroup analyses for men and women, as well as for participants of different age groups. We were able to show that, aside from external demands, there were no influences of sex and age on changes in stress and mental health problems as well as on increases going along with clinically significant stress and symptom levels during lockdown. This suggests a generalizability of our results to male participants, and participants of different age groups. Furthermore, there was an overrepresentation of participants with a university and university of applied sciences degree, and an underrepresentation of participants with a vocational training or a master/technician degree and without a professional qualification in comparison to the distribution of professional qualifications in the German general population as published by the German Federal Statistical Office [76]. However, we registered participants with different sociodemographic characteristics (Table 1), and internal validity was ensured by observing changes experienced during first COVID-19 lockdown in comparison to before the pandemic at the individual level, instead of comparing population prevalence or sample means from different samples. Furthermore, the bounded region of Southeastern Germany, the short data collection period during first COVID-19 lockdown, and the exclusion of participants affected by a COVID-19 infection or quarantine constituted a relatively simultaneous experience in all participants. To control for differences in sociodemographic, health-, and pandemic-related variables, we furthermore reported on sex, age, relationship status, professional qualification, mental health treatment, chronic physical diseases, employment status, and pandemic-related conditions like changes in employment status, fear of losing one’s livelihood, the current work setting (e.g., home-office), childcare responsibilities, social contact, and COVID-19-related fears; all previously found to be associated with worse mental health during the pandemic [for examples see [63, 77–81]].

Finally, we did not analyze the influence of all those variables on perceived increases or decreases in different subareas of stress and mental health problems in our sample, but only focused on the influence of sex and age. Thus, additional research is needed to target further potential risk and protection factors, also in our sample. For example, social support and personality factors seem of great interest in this context. Moreover, it would be interesting to disentangle the contribution of different aspects of the pandemic lockdown to increases in stress and mental health problems, like of social isolation, of fears of an infection with SARS-CoV-2 and illness with COVID-19, or of fears of sanctions for a violation of protection measures. That we also did not consider alternative explanations for changes in stress and mental health during first COVID-19 lockdown, like life-events occurring independently from the pandemic, represents a further limitation of our study. However, since the time phase of first lockdown was narrow, we do not expect this limitation to have severely affected our results.

## Conclusion

This study on how adults from Southeastern Germany feel they have changed from before the COVID-19 pandemic to the first lockdown showed a dissociation of perceived changes in subareas of stress and mental health. Our findings suggest that experiencing an increase in depressive symptoms and general stress reactions might represent a more typical response to the early phase of the pandemic and the associated lockdown than others (like experiencing increases in specific PTSD symptoms, symptoms of acute fear and panic, pathological worrying, and health anxiety not specifically related to COVID), while demands as an external stressor might even be predominantly decreased. However, this perceived decrease in demands might be more common in younger adults, while midadulthood and female sex was associated with a higher frequency of experiencing an increase in external demands during first lockdown. Although the sample cannot be considered as representative for the general population of Southeastern Germany, and the assessment of how people feel they have changed does not unconditionally indicate true change, our exploratory results on adults affected by strict stay-at-home orders and social contact prohibitions during first COVID-19 lockdown in Southeastern Germany provide the first comparison between individually perceived changes in multiple outcome measures. Our findings suggest that different subareas of perceived stress and mental health problems do not equally worsen during a pandemic lockdown, indicating the need for a more differentiated view of the impact of the COVID-19 pandemic and associated lockdown on mental health.

The considerable proportion of individuals perceiving increases in depressive and general stress symptoms and showing a symptom severity of clinical relevance during lockdown indicates a need for additional health care capacity during the pandemic. We suggest targeted interventions and prevention strategies for frequently affected symptoms. Future studies should investigate the dissociation of changes in different subareas of perceived stress and mental health problems in representative samples, to verify the findings and derived recommendations concerning mental health care. Furthermore, the maintenance of perceived stress and mental health problems beyond first lockdown must be examined in follow-up measures. Future studies should also shed light on associations between lockdown and living conditions and changes in specific subareas of perceived stress and mental health, on risk- and resilience factors, changes in further mental health problems (e.g., social phobia, obsessive-compulsive or eating disorder), and in biological markers associated with mental health problems.

## Supplementary Information

**Additional file 1:** **Figure S1.**

**Additional file 2:** **Table S1.**

**Additional file 3:** **Table S2.**

**Additional file 4:** **Table S3.**

**Additional file 5:** **Table S4.**

**Additional file 6:** **Table S5.**

**Additional file 7:** **Table S6.**

**Additional file 8:** **Text 1.**

## Data Availability

The dataset analyzed during the current study is available in the OSF repository, https://osf.io/emdv4/?view_only=295744832f934170bdd09beaceb53f7f.

## Abbreviation

CBC anxiety: Child Behaviour Checklist Anxiety
CI: Confidence Interval
CRS: Clinical Reference Sample
DASS21: 21-item Depression-Anxiety-Stress-Scales
E.g.: exempli gratia (for example)
GAD: Generalized Anxiety Disorder
GAD-2: Generalized Anxiety Disorder 2-item
GAD-7: Generalized Anxiety Disorder Scale-7
HADS: Hospital Anxiety and Depression Scale
M: Mean
MK-HAI: German-modified Health-Anxiety-Inventory
p: p-value
PHQ-D: Patient Health Questionnaire (German)
PROMIS: Patient-reported Outcomes Measurement Information System
ps, p-values PSQ-20: Perceived-Stress-Questionnaire 20-items-version
PSWQ-PW: Penn-State Worry-Questionnaire Past-Week
PTSD: Post-Traumatic Stress Disorder
PTSS-10: 10 item Post-Traumatic Symptom Scale
SAS: Self-Rating Anxiety Scale
SD: Standard Deviation
STROBE: STrengthening the Reporting of OBservational studies in Epidemiology
