# Supplementary material for: Individual changes in stress, depression, anxiety, pathological worry, posttraumatic stress, and health anxiety from before to during the COVID-19 pandemic in adults from Southeastern Germany
Source: BMC Psychiatry. 2022 Aug 5;22:528. doi: 10.1186/s12888-022-04148-y (PMC9354380; doi:10.1186/s12888-022-04148-y)
Supplement: Supplementary file 2 — Additional file 2: Table S1. [file 12888_2022_4148_MOESM2_ESM.pdf]

Additional Table 1. Comprehensive outcome data and results for perceived stress.

		Change category subsamples[a]						
		Decrease				Increase		
Variable	Total sample	Strong	Moderate	Overall	No change	Overall	Moderate	Strong
**Perceived stress**								
n (%)[b]	396 (100.0)	5 (1.3)	55 (13.9)	60 (15.2)	233 (58.8)	103 (26.0)	97 (24.5)	6 (1.5)
Score, *M* (*SD*)[c]	39.66 (21.44)	27.67 (32.09)	24.12 (15.74)	24.42 (17.25)	34.34 (17.42)	60.57 (16.62)	59.14 (15.93)	83.61 (9.09)
n (%) c.r. within cs[d]	–	1 (20.0)	5 (9.1)	6 (10.0)	57 (24.5)	80 (77.7)	74 (76.3)	6 (100.0)
n (%) c.r. within ts[d]	143 (36.1)	1 (0.3)	5 (1.3)	6 (1.5)	57 (14.4)	80 (20.2)	74 (18.7)	6 (1.5)
Worries								
n (%)[b]	396 (100.0)	3 (0.8)	29 (7.3)	32 (8.1)	224 (56.6)	140 (35.4)	119 (30.1)	21 (5.3)
Score, *M* (*SD*)[c]	36.75 (25.78)	46.67 (48.07)	23.22 (19.16)	25.42 (23.0)	26.37 (19.23)	55.95 (24.54)	51.93 (23.35)	78.73 (18.09)
n (%) c.r. within cs[d]	–	1 (33.3)	4 (13.8)	5 (15.6)	39 (17.4)	96 (68.6)	75 (63.0)	21 (100.0)
n (%) c.r. within ts[d]	140 (35.4)	1 (0.3)	4 (1.0)	5 (1.3)	39 (9.8)	96 (24.2)	75 (18.9)	21 (5.3)
Tension								
n (%)[b]	396 (100.0)	15 (3.8)	51 (12.9)	66 (16.7)	203 (51.3)	127 (32.1)	102 (25.8)	25 (6.3)
Score, *M* (*SD*)[c]	40.76 (27.02)	14.22 (21.06)	23.79 (20.19)	21.62 (20.62)	32.38 (21.51)	64.09 (21.76)	59.87 (21.29)	81.33 (13.88)
n (%) c.r. within cs[d]	–	1 (6.7)	7 (13.7)	8 (12.1)	59 (29.1)	104 (81.9)	79 (77.5)	25 (100.0)
n (%) c.r. within ts[d]	171 (43.2)	1 (0.3)	7 (1.8)	8 (2.0)	59 (14.9)	104 (26.3)	79 (19.9)	25 (6.3)
Joy								
n (%)[b]	396 (100.0)	26 (6.6)	125 (31.6)	151 (38.1)	205 (51.8)	40 (10.1)	35 (8.8)	5 (1.3)
Score, *M* (*SD*)[c]	52.61 (23.89)	16.67 (11.04)	42.19 (19.19)	37.79 (20.45)	60.59 (21.32)	67.67 (19.42)	68.95 (18.00)	58.67 (28.44)
n (%) c.r. within cs[d]	–	26 (100.0)	68 (54.4)	94 (62.3)	43 (21.0)	4 (10.0)	3 (8.6)	1 (20.0)
n (%) c.r. within ts[d]	141 (35.6)	26 (6.6)	68 (17.2)	94 (23.7)	43 (10.9)	4 (1.0)	3 (0.8)	1 (0.3)
Demands								
n (%)[b]	396 (100.0)	51 (12.9)	104 (26.3)	155 (39.1)	169 (42.7)	72 (18.2)	57 (14.4)	15 (3.8)
Score, *M* (*SD*)[c]	33.74 (25.31)	11.76 (15.92)	22.05 (17.53)	18.67 (17.64)	34.75 (20.59)	63.80 (21.69)	58.25 (18.50)	84.89 (20.39)
n (%) c.r. within cs[d]	–	3 (5.9)	13 (12.5)	16 (10.3)	57 (33.7)	59 (81.9)	45 (78.9)	14 (93.3)
n (%) c.r. within ts[d]	132 (33.3)	3 (0.8)	13 (3.3)	16 (4.0)	57 (14.4)	59 (14.9)	45 (11.4)	14 (3.5)

c.r., clinically relevant; ts, total sample; cs, change category subsample.

[a] An adapted version of PSQ-20 (Perceived-Stress-Questionnaire) was used to measure changes in perceived stress during lockdown in comparison to before the COVID-19 pandemic on item level (–2 *much less than before corona*; +2 *much more than before corona*). Change indices (–2 *strong decrease* to +2 *strong increase*) were calculated for each participant and each outcome variable by averaging the change values of the total questionnaire items or the items of the respective subscale, and were grouped into three change categories (–2.00 to –1.50 *decrease* [overall]; –0.49 to +0.49 *no change*; +0.50 to +2.00 *increase* [overall]), and additionally into five change categories (–2.00 to –1.50 *strong decrease*; –1.49 to –0.50 *moderate decrease*; –0.49 to +0.49 *no change*; +0.50 to +1.49 *moderate increase*; +1.50 to +2.00 *strong increase*).

[b] Absolute and relative frequencies of the total sample and of participants experiencing a strong decrease, moderate decrease, decrease (overall), no change, increase (overall), moderate increase, or strong increase in total perceived stress or the respective subscale during first lockdown in comparison to before the COVID-19 pandemic.

[c] Questionnaire scores for PSQ-20 total, and the subscales worries, tension, joy, and demands (each range 0–100), assessing the participants' state during lockdown.

[d] Number and percentage (within change category subsample [cs] or within total sample [ts]) of participants with clinically relevant questionnaire scores during lockdown according to criterion c thresholds calculated with reference to clinical samples (≥46.37 for PSQ-20 total, ≥44.84 for PSQ-20 worries, ≥45.77 for PSQ-20 tension, ≥44.66 for PSQ-20 joy, ≥40.02 for PSQ-20 demands).
