# Supplementary material for: Individual changes in stress, depression, anxiety, pathological worry, posttraumatic stress, and health anxiety from before to during the COVID-19 pandemic in adults from Southeastern Germany
Source: BMC Psychiatry. 2022 Aug 5;22:528. doi: 10.1186/s12888-022-04148-y (PMC9354380; doi:10.1186/s12888-022-04148-y)
Supplement: Supplementary file 3 — Additional file 3: Table S2. [file 12888_2022_4148_MOESM3_ESM.pdf]

Additional Table 2. Comprehensive outcome data and results for mental health problems.

		Change category subsamples[a]						
		Decrease				Increase		
	Total sample	Strong	Moderate	Overall	No change	Overall	Moderate	Strong
**Depression**								
n (%)[b]	396 (100.0)	2 (0.5)	12 (3.0)	14 (3.5)	279 (70.5)	103 (26.0)	84 (21.2)	19 (4.8)
Score, *M* (*SD*)[c]	4.68 (4.70)	2.00 (1.41)	6.17 (6.04)	5.57 (5.77)	2.94 (3.20)	9.29 (4.84)	8.30 (4.42)	13.68 (4.18)
n (%) c.r. within cs[d]	–	0 (0.0)	2 (16.7)	2 (14.3)	12 (4.3)	41 (39.8)	24 (28.6)	17 (89.5)
n (%) c.r. within ts[d]	55 (13.9)	0 (0.0)	2 (0.5)	2 (0.5)	12 (3.0)	41 (10.4)	24 (6.1)	17 (4.3)
**Posttraumatic stress**								
n (%)[b]	396 (100.0)	1 (0.3)	11 (2.8)	12 (3.0)	283 (71.5)	101 (25.5)	96 (24.2)	5 (1.3)
Score, *M* (*SD*)[c]	19.57 (12.88)	56.00[e]	15.91 (11.10)	19.25 (15.69)	15.59 (10.59)	30.75 (11.87)	30.00 (11.55)	45.20 (8.79)
n (%) c.r. within cs[d]	–	1 (100.0)	2 (18.2)	3 (25.0)	66 (23.3)	73 (72.3)	68 (70.8)	5 (100.0)
n (%) c.r. within ts[d]	142 (35.9)	1 (0.3)	2 (0.5)	3 (0.8)	66 (16.7)	73 (18.4)	68 (17.2)	5 (1.3)
**Anxiety**								
n (%)[b]	396 (100.0)	4 (1.0)	5 (1.3)	9 (2.3)	365 (92.2)	22 (5.6)	19 (4.8)	3 (0.8)
Score, *M* (*SD*)[c]	2.16 (2.90)	1.00 (1.15)	5.00 (5.57)	3.22 (4.52)	1.76 (2.28)	8.36 (4.08)	7.53 (3.36)	13.67 (4.93)
n (%) c.r. within cs[d]	–	0 (0.00)	2 (40.0)	2 (22.2)	22 (6.0)	18 (81.8)	15 (78.9)	3 (100.0)
n (%) c.r. within ts[d]	42 (10.6)	0 (0.0)	2 (0.5)	2 (0.5)	22 (5.6)	18 (4.5)	15 (3.8)	3 (0.8)
**Pathological worry**								
n (%)[b]	396 (100.0)	1 (0.3)	10 (2.5)	11 (2.8)	346 (87.4)	39 (9.8)	38 (9.6)	1 (0.3)
Score, *M* (*SD*)[c]	37.83 (18.73)	59.00[e]	34.20 (15.48)	36.45 (16.48)	35.51 (17.81)	58.82 (13.88)	58.82 (14.07)	59.00[e]
n (%) c.r. within cs[d]	–	1 (100.0)	1 (10.0)	2 (18.2)	54 (15.6)	27 (69.2)	26 (68.4)	1 (100.0)
n (%) c.r. within ts[d]	83 (21.0)	1 (0.3)	1 (0.3)	2 (0.5)	54 (13.6)	27 (6.8)	26 (6.6)	1 (0.3)
**Health anxiety**								
n (%)[b]	396 (100.0)	0 (0.0)	3 (0.8)	3 (0.8)	364 (91.9)	29 (7.3)	28 (7.1)	1 (0.3)
Score, *M* (*SD*)[c]	17.19 (11.07)	–	23.33 (6.66)	23.33 (6.66)	15.86 (10.24)	33.28 (8.39)	32.57 (7.62)	53.00[e]
n (%) c.r. within cs[d]	–	0 (0.0)	1 (33.3)	1 (33.3)	86 (23.6)	26 (89.7)	25 (89.3)	1 (100.00)
n (%) c.r. within ts[d]	113 (28.5)	0 (0.0)	1 (0.3)	1 (0.3)	86 (21.7)	26 (6.6)	25 (6.3)	1 (0.3)

c.r., clinically relevant; ts, total sample; cs, change category subsample.

[a] Adapted versions of the original questionnaires stated below were used to measure changes in mental health problems during lockdown in comparison to before the COVID-19 pandemic on item level (–2 *much less than before corona*; +2 *much more than before corona*). Change indices (–2 *strong decrease* to +2 *strong increase*) were calculated for each participant and each outcome variable by averaging the change values for the respective questionnaire items, and were grouped into three change categories (–2.00 to –1.50 *decrease* [overall]; –0.49 to +0.49 *no change*; +0.50 to +2.00 *increase* [overall]), and additionally into five change categories (–2.00 to –1.50 *strong decrease*; –1.49 to –0.50 *moderate decrease*; –0.49 to +0.49 *no change*; +0.50 to +1.49 *moderate increase*; +1.50 to +2.00 *strong increase*).

[b] Absolute and relative frequencies of the total sample and of participants experiencing a strong decrease, moderate decrease, decrease (overall), no change, increase (overall), moderate increase, or strong increase in the respective mental health problems during first lockdown in comparison to before the COVID-19 pandemic.

[c] Original questionnaire scores for DASS21 (Depression-Anxiety-Stress-Scales) subscales depression and anxiety (each range 0–21), PTSS-10 (Posttraumatic-Symptom-Scale; range 0–60), PSWQ-PW (Penn-State-Worry-Questionnaire-Past-Week; range 0–90), and MK-HAI (German-modified-Health-Anxiety-Inventory; range 0–56), assessing the participants' state during lockdown.

[d] Number and percentage (within change category sample [cs] or within total sample [ts]) of participants with clinically relevant questionnaire scores during lockdown according to cut-offs stated by the questionnaire authors (≥10 for DASS21 depression, ≥24 for PTSS-10, and ≥6 for DASS21 anxiety), or according to criterion c thresholds calculated with reference to clinical samples (≥54.54 for PSWQ-PW, and ≥23.93 for MK-HAI).
[e] If there was only one participant grouped into a change category, the absolute questionnaire score of this participant is stated instead of *M* (*S.D.*) over all participants of the respective change category.
