# Supplementary material for: Individual changes in stress, depression, anxiety, pathological worry, posttraumatic stress, and health anxiety from before to during the COVID-19 pandemic in adults from Southeastern Germany
Source: BMC Psychiatry. 2022 Aug 5;22:528. doi: 10.1186/s12888-022-04148-y (PMC9354380; doi:10.1186/s12888-022-04148-y)
Supplement: Supplementary file 4 — Additional file 4: Table S3. [file 12888_2022_4148_MOESM4_ESM.pdf]

Additional Table 3. Associations between the participants' sex and changes in stress and mental health problems during lockdown.

Variables		Sex						χ^2-test	
		male		female		total		χ^2	*p*
		n	%	*n*	%	*n*	%		
**Perceived stress**									
Total stress	decrease	17_a	14.7%	43_a	15.5%	60	15.2%	2.11	.341
	no change	74_a	63.8%	157_a	56.5%	231	58.6%		
	increase	25_a	21.6%	78_a	28.1%	103	26.1%		
	total	116	100.0%	278	100.0%	394	100.0%		
Worries	decrease	7_a	6.0%	25_a	9.0%	32	8.1%	1.80	.427
	no change	71_a	61.2%	152_a	54.7%	223	56.6%		
	increase	38_a	32.8%	101_a	36.3%	139	35.3%		
	total	116	100.0%	278	100.0%	394	100.0%		
Tension	decrease	19_a	16.4%	47_a	16.9%	66	16.8%	6.20	.046
	no change	70_a	60.3%	133_b	47.8%	203	51.5%		
	increase	27_a	23.3%	98_b	35.3%	125	31.7%		
	total	116	100.0%	278	100.0%	394	100.0%		
Joy	decrease	41_a	35.3%	108_a	38.8%	149	37.8%	0.51	.793
	no change	62_a	53.4%	143_a	51.4%	205	52.0%		
	increase	13_a	11.2%	27_a	9.7%	40	10.2%		
	total	116	100.0%	278	100.0%	394	100.0%		
Demands	decrease	42_a	36.2%	111_a	39.9%	153	38.8%	14.45	**.001**
	no change	64_a	55.2%	105_b	37.8%	169	42.9%		
	increase	10_a	8.6%	62_b	22.3%	72	18.3%		
	total	116	100.0%	278	100.0%	394	100.0%		
**Mental health problems**									
Depression	decrease	3_a	2.6%	11_a	4.0%	14	3.6%	3.92	.142
	no change	90_a	77.6%	188_b	67.6%	278	70.6%		
	increase	23_a	19.8%	79_a	28.4%	102	25.9%		
	total	116	100.0%	278	100.0%	394	100.0%		
PTSD	decrease	4_a	3.4%	8_a	2.9%	12	3.0%	8.07	.019
	no change	94_a	81.0%	189_b	68.0%	283	71.8%		
	increase	18_a	15.5%	81_b	29.1%	99	25.1%		
	total	116	100.0%	278	100.0%	394	100.0%		
Anxiety	decrease	2_a	1.7%	7_a	2.5%	9	2.3%	4.96	.074
	no change	112_a	96.6%	251_b	90.3%	363	92.1%		
	increase	2_a	1.7%	20_b	7.2%	22	5.6%		
	total	116	100.0%	278	100.0%	394	100.0%		
Pathological Worry	decrease	4_a	3.4%	7_a	2.5%	11	2.8%	5.52	.061
	no change	107_a	92.2%	238_a	85.6%	345	87.6%		
	increase	5_a	4.3%	33_b	11.9%	38	9.6%		
	total	116	100.0%	278	100.0%	394	100.0%		
Health Anxiety	decrease	0_a	0.0%	3_a	1.1%	3	0.8%	1.72	.486
	no change	109_a	94.0%	253_a	91.0%	362	91.9%		
	increase	7_a	6.0%	22_a	7.9%	29	7.4%		
	total	116	100.0%	278	100.0%	394	100.0%		

The cross table displays the absolute and relative frequencies of participants experiencing a decrease, no change, or increase within the total sample, and within men and women, separately. Furthermore, the table displays results of comparisons of frequencies for decreases, no changes, an increases between men and women using χ^2-tests. Significant *p*-values (alpha-level=.001) are indicated in bold letters.
Adapted versions of the PSQ-20 (Perceived-Stress-Questionnaire), DASS21 (Depression-Anxiety-Stress-Scales) subscales depression and anxiety, PTSS-10 (Posttraumatic-Symptom-Scale), PSWQ-PW (Penn-State-Worry-Questionnaire-Past-Week), and MK-HAI (German-modified-Health-Anxiety-Inventory) were used to measure changes in perceived stress and mental health problems during lockdown in comparison to before the COVID-19 pandemic on item level (–2 *much less than before corona*; +2 *much more than before corona*). Change indices (–1 *strong decrease* to +2 *strong increase*) were calculated for each participant and each outcome variable by averaging the change values of the total questionnaire items or the items of the respective subscale, and were grouped into three change categories (–2.00 to –1.50 *decrease*; –0.49 to +0.49 *no change*; +0.50 to +2.00 *increase*).
$_{a/b}$: Subletters indicate significantly different proportions within one row, indicated by z-tests.
