# Supplementary material for: Individual changes in stress, depression, anxiety, pathological worry, posttraumatic stress, and health anxiety from before to during the COVID-19 pandemic in adults from Southeastern Germany
Source: BMC Psychiatry. 2022 Aug 5;22:528. doi: 10.1186/s12888-022-04148-y (PMC9354380; doi:10.1186/s12888-022-04148-y)
Supplement: Supplementary file 5 — Additional file 5: Table S4. [file 12888_2022_4148_MOESM5_ESM.pdf]

Additional Table 4. Associations between the participants’ sex and clinically relevant increases in stress and mental health problems during lockdown.

		Sex						χ^2-test	
		male		female		total		χ^2	*p*
Variables		*n*	%	*n*	%	*n*	%		
**Participants with increases in perceived stress**									
Total stress	not clin. rel.	3$_a$	12.0%	20$_a$	25.6%	23	22.3%	2.03	.180
	clin. rel.	22$_a$	88.0%	58$_a$	74.4%	80	77.7%		
	total	25	100.0%	78	100.0%	103	100.0%		
Worries	not clin. rel.	13$_a$	34.2%	30$_a$	29.7%	43	30.9%	0.26	.682
	clin. rel.	25$_a$	65.8%	71$_a$	70.3%	96	69.1%		
	total	38	100.0%	101	100.0%	139	100.0%		
Tension	not clin. rel.	3$_a$	11.1%	19$_a$	19.4%	22	17.6%	1.00	.402
	clin. rel.	24$_a$	88.9%	79$_a$	80.6%	103	82.4%		
	total	27	100.0%	98	100.0%	125	100.0%		
Joy	not clin. rel.	16$_a$	39.0%	40$_a$	37.0%	56	37.6%	0.05	.851
	clin. rel.	25$_a$	61.0%	68$_a$	63.0%	93	62.4%		
	total	41	100.0%	108	100.0%	149	100.0%		
Demands	not clin. rel.	0$_a$	0.0%	13$_a$	21.0%	13	18.1%	2.56	.191
	clin. rel.	10$_a$	100.0%	49$_a$	79.0%	59	81.9%		
	total	10	100.0%	62	100.0%	72	100.0%		
**Participants with increases in mental health problems**									
Depression	not clin. rel.	15$_a$	65.2%	47$_a$	59.5%	62	60.8%	0.25	.639
	clin. rel.	8$_a$	34.8%	32$_a$	40.5%	40	39.2%		
	total	23	100.0%	79	100.0%	102	100.0%		
PTSD	not clin. rel.	10$_a$	55.6%	16$_b$	19.8%	26	26.3%	9.75	.003
	clin. rel.	8$_a$	44.4%	65$_b$	80.2%	73	73.7%		
	total	18	100.0%	81	100.0%	99	100.0%		
Anxiety	not clin. rel.	1$_a$	50.0%	3$_a$	15.0%	4	18.2%	1.50	.338
	clin. rel.	1$_a$	50.0%	17$_a$	85.0%	18	81.8%		
	total	2	100.0%	20	100.0%	22	100.0%		
Path. Worry	not clin. rel.	4$_a$	80.0%	7$_b$	21.2%	11	28.9%	7.30	.019
	clin. rel.	1$_a$	20.0%	26$_b$	78.8%	27	71.1%		
	total	5	100.0%	33	100.0%	38	100.0%		
Health Anxiety	not clin. rel.	0$_a$	0.0%	3$_a$	13.6%	3	10.3%	1.07	.557
	clin. rel.	7$_a$	100.0%	19$_a$	86.4%	26	89.7%		
	total	7	100.0%	22	100.0%	29	100.0%		

The cross table displays the absolute and relative frequencies of participants reporting an increase in stress or mental health problems from before to during lockdown according to our change measurement, and showing a clinically relevant or not clinically relevant questionnaire score for those stress or mental health problems during lockdown according to our state measurement. The absolute and relative frequencies are displayed for the whole sample, and separately for men and women. Furthermore, the table displays results of comparisons of frequencies for clinically relevant and not clinically relevant increases between men and women using χ^2-tests. The alpha-level for statistical significance was set to .001.

Adapted versions of the PSQ-20 (Perceived-Stress-Questionnaire), DASS21 (Depression-Anxiety-Stress-Scales) subscales depression and anxiety, PTSS-10 (Posttraumatic-Symptom-Scale), PSWQ-PW (Penn-State-Worry-Questionnaire-Past-Week), and MK-HAI (German-modified-Health-Anxiety-Inventory) were used to measure changes in perceived stress and mental health problems during lockdown in comparison to before the COVID-19 pandemic on item level (–2 *much less than before corona*; +2 *much more than before corona*). Change indices (–1 *strong decrease* to +2 *strong increase*) were calculated for each participant and each outcome variable by averaging the change values of the total questionnaire items or the items of the respective subscale, and were grouped into three change categories (–2.00 to –1.50 *decrease*; –0.49 to +0.49 *no change*; +0.50 to +2.00 *increase*). The original questionnaires PSQ-20 total, with the subscales worries, tension, joy, and demands, DASS21 subscales depression and anxiety, PTSS-10, PSWQ-PW, and MK-HAI were used to assess the participants’ state during lockdown. Clinically relevant questionnaire scores were defined according to cut-offs stated by the questionnaire authors (≥10 for DASS21 depression, ≥24 for PTSS-10, and ≥6 for DASS21 anxiety), or according to criterion c thresholds calculated with reference to clinical samples (≥54.54 for PSWQ-PW, ≥23.93 for MK-HAI, and ≥46.37 for PSQ-20 total, ≥44.84 for PSQ-20 worries, ≥45.77 for PSQ-20 tension, ≥44.66 for PSQ-20 joy, ≥40.02 for PSQ-20 demands).

clin.rel., clinically relevant. path., pathological.

$_{a/b}$: Subletters indicate significantly different proportions within one row, indicated by z-tests.
