# Supplementary material for: Individual changes in stress, depression, anxiety, pathological worry, posttraumatic stress, and health anxiety from before to during the COVID-19 pandemic in adults from Southeastern Germany
Source: BMC Psychiatry. 2022 Aug 5;22:528. doi: 10.1186/s12888-022-04148-y (PMC9354380; doi:10.1186/s12888-022-04148-y)
Supplement: Supplementary file 6 — Additional file 6: Table S5. [file 12888_2022_4148_MOESM6_ESM.pdf]

Additional Table 5. Associations between the participants' age and changes in stress and mental health problems during lockdown.

Variables		Age group								χ^2-test	
		≤30 years		31–50 years		>50 years		total		χ^2	*p*
		n	%	*n*	%	*n*	%	*n*	%		
**Perceived stress**											
Total stress	decrease	27_{a}	19.6%	17_{b}	11.0%	$16_{a, b}$	15.5%	60	15.2%	10.94	.027
	no change	83_{a}	60.1%	85_{a}	54.8%	65_{a}	63.1%	233	58.8%		
	increase	28_{a}	20.3%	53_{b}	34.2%	22_{a}	21.4%	103	26.0%		
	total	138	100.0%	155	100.0%	103	100.0%	396	100.0%		
Worries	decrease	13_{a}	9.4%	9_{a}	5.8%	10_{a}	9.7%	32	8.1%	6.09	.193
	no change	70_{a}	50.7%	89_{a}	57.4%	65_{a}	63.1%	224	56.6%		
	increase	55_{a}	39.9%	$57_{a, b}$	36.8%	28_{b}	27.2%	140	35.4%		
	total	138	100.0%	155	100.0%	103	100.0%	396	100.0%		
Tension	decrease	32_{a}	23.2%	$23_{a, b}$	14.8%	11_{b}	10.7%	66	16.7%	12.94	.011
	no change	65_{a}	47.1%	73_{a}	47.1%	65_{b}	63.1%	203	51.3%		
	increase	$41_{a, b}$	29.7%	59_{b}	38.1%	27_{a}	26.2%	127	32.1%		
	total	138	100.0%	155	100.0%	103	100.0%	396	100.0%		
Joy	decrease	53_{a}	38.4%	63_{a}	40.6%	35_{a}	34.0%	151	38.1%	4.84	.307
	no change	66_{a}	47.8%	79_{a}	51.0%	60_{a}	58.3%	205	51.8%		
	increase	19_{a}	13.8%	13_{a}	8.4%	8_{a}	7.8%	40	10.1%		
	total	138	100.0%	155	100.0%	103	100.0%	396	100.0%		
Demands	decrease	73_{a}	52.9%	48_{b}	31.0%	34_{b}	33.0%	155	39.1%	31.06	**<.001**
	no change	53_{a}	38.4%	62_{a}	40.0%	54_{b}	52.4%	169	42.7%		
	increase	12_{a}	8.7%	45_{b}	29.0%	15_{a}	14.6%	72	18.2%		
	total	138	100.0%	155	100.0%	103	100.0%	396	100.0%		
**Mental health problems**											
Depression	decrease	10_{a}	7.2%	2_{b}	1.3%	$2_{a, b}$	1.9%	14	3.5%	10.82	.027
	no change	89_{a}	64.5%	$111_{a, b}$	71.6%	79_{b}	76.7%	279	70.5%		
	increase	39_{a}	28.3%	42_{a}	27.1%	22_{a}	21.4%	103	26.0%		
	total	138	100.0%	155	100.0%	103	100.0%	396	100.0%		
PTSD	decrease	6_{a}	4.3%	5_{a}	3.2%	1_{a}	1.0%	12	3.0%	13.85	.007
	no change	92_{a}	66.7%	103_{a}	66.5%	88_{b}	85.4%	283	71.5%		
	increase	40_{a}	29.0%	47_{a}	30.3%	14_{b}	13.6%	101	25.5%		
	total	138	100.0%	155	100.0%	103	100.0%	396	100.0%		
Anxiety	decrease	5_{a}	3.6%	1_{a}	0.6%	3_{a}	2.9%	9	2.3%	3.86	.435
	no change	124_{a}	89.9%	147_{a}	94.8%	94_{a}	91.3%	365	92.2%		
	increase	9_{a}	6.5%	7_{a}	4.5%	6_{a}	5.8%	22	5.6%		
	total	138	100.0%	155	100.0%	103	100.0%	396	100.0%		
Path. Worry	decrease	7_{a}	5.1%	$4_{a, b}$	2.6%	0_{b}	0.0%	11	2.8%	10.76	.026
	no change	113_{a}	81.9%	135_{a}	87.1%	98_{b}	95.1%	346	87.4%		
	increase	18_{a}	13.0%	$16_{a, b}$	10.3%	5_{b}	4.9%	39	9.8%		
	total	138	100.0%	155	100.0%	103	100.0%	396	100.0%		
Health Anxiety	decrease	2_{a}	1.4%	1_{a}	0.6%	0_{a}	0.0%	3	0.8%	3.46	.503
	no change	124_{a}	89.9%	146_{a}	94.2%	94_{a}	91.3%	364	91.9%		
	increase	12_{a}	8.7%	8_{a}	5.2%	9_{a}	8.7%	29	7.3%		
	total	138	100.0%	155	100.0%	103	100.0%	396	100.0%		

The cross table displays the absolute and relative frequencies of participants experiencing a decrease, no change, or increase within the total sample, and within different age groups, separately. Furthermore, the table displays results of comparisons of frequencies for decreases, no changes, an increases between different age groups using χ^2-tests. Significant *p*-values (alpha-level=.001) are indicated in bold letters.
Adapted versions of the PSQ-20 (Perceived-Stress-Questionnaire), DASS21 (Depression-Anxiety-Stress-Scales) subscales depression and anxiety, PTSS-10 (Posttraumatic-Symptom-Scale), PSWQ-PW (Penn-State-Worry-Questionnaire-Past-Week), and MK-HAI (German-modified-Health-Anxiety-Inventory) were used to measure changes in perceived stress and mental health problems during lockdown in comparison to before the COVID-19 pandemic on item level (–2 *much less than before corona*; +2 *much more than before corona*). Change indices (–1 *strong decrease* to +2 *strong increase*) were calculated for each participant and each outcome variable by averaging the change values of the total questionnaire items or the items of the respective subscale, and were grouped into three change categories (–2.00 to –1.50 *decrease*; –0.49 to +0.49 *no change*; +0.50 to +2.00 *increase*).
$_{a/b}$: Subletters indicate significantly different proportions within one row, indicated by z-tests.
