# Supplementary material for: Individual changes in stress, depression, anxiety, pathological worry, posttraumatic stress, and health anxiety from before to during the COVID-19 pandemic in adults from Southeastern Germany
Source: BMC Psychiatry. 2022 Aug 5;22:528. doi: 10.1186/s12888-022-04148-y (PMC9354380; doi:10.1186/s12888-022-04148-y)
Supplement: Supplementary file 7 — Additional file 7: Table S6. [file 12888_2022_4148_MOESM7_ESM.pdf]

Additional Table 6. Associations between the participants' age and clinically relevant increases in stress and mental health problems during lockdown.

Variables		Age group								χ^2-test	
		≤30		31–50		>50		total		χ^2	*p*
		n	*%*	*n*	*%*	*n*	*%*	*n*	*%*		
**Participants with increases in perceived stress**											
Total stress	not clin. rel.	6_a	21.4%	10_a	18.9%	7_a	31.8%	23	22.3%	1.52	.497
	clin. rel.	22_a	78.6%	43_a	81.1%	15_a	68.2%	80	77.7%		
	total	28	100.0%	53	100.0%	22	100.0%	103	100.0%		
Worries	not clin. rel.	12_a	21.8%	21_a	36.8%	11_a	39.3%	44	31.4%	3.93	.132
	clin. rel.	43_a	78.2%	36_a	63.2%	17_a	60.7%	96	68.6%		
	total	55	100.0%	57	100.0%	28	100.0%	140	100.0%		
Tension	not clin. rel.	10_a	24.4%	7_a	11.9%	6_a	22.2%	23	18.1%	2.95	.259
	clin. rel.	31_a	75.6%	52_a	88.1%	21_a	77.8%	104	81.9%		
	total	41	100.0%	59	100.0%	27	100.0%	127	100.0%		
Joy	not clin. rel.	22_a	41.5%	21_a	33.3%	14_a	40.0%	57	37.7%	0.92	.664
	clin. rel.	31_a	58.5%	42_a	66.7%	21_a	60.0%	94	62.3%		
	total	53	100.0%	63	100.0%	35	100.0%	151	100.0%		
Demands	not clin. rel.	2_a	16.7%	6_a	13.3%	5_a	33.3%	13	18.1%	3.06	.276
	clin. rel.	10_a	83.3%	39_a	86.7%	10_a	66.7%	59	81.9%		
	total	12	100.0%	45	100.0%	15	100.0%	72	100.0%		
**Participants with increases in mental health problems**											
Depression	not clin. rel.	18_a	46.2%	31_b	73.8%	$13_{a, b}$	59.1%	62	60.2%	6.47	.041
	clin. rel.	21_a	53.8%	11_b	26.2%	$9_{a, b}$	40.9%	41	39.8%		
	total	39	100.0%	42	100.0%	22	100.0%	103	100.0%		
PTSD	not clin. rel.	10_a	25.0%	15_a	31.9%	3_a	21.4%	28	27.7%	0.84	.716
	clin. rel.	30_a	75.0%	32_a	68.1%	11_a	78.6%	73	72.3%		
	total	40	100.0%	47	100.0%	14	100.0%	101	100.0%		
Anxiety	not clin. rel.	1_a	11.1%	3_a	42.9%	0_a	0.0%	4	18.2%	4.50	.140
	clin. rel.	8_a	88.9%	4_a	57.1%	6_a	100.0%	18	81.8%		
	total	9	100.0%	7	100.0%	6	100.0%	22	100.0%		
Path. Worry	not clin. rel.	6_a	33.3%	4_a	25.0%	2_a	40.0%	12	30.8%	0.51	.801
	clin. rel.	12_a	66.7%	12_a	75.0%	3_a	60.0%	27	69.2%		
	total	18	100.0%	16	100.0%	5	100.0%	39	100.0%		
Health Anxiety	not clin. rel.	2_a	16.7%	1_a	12.5%	0_a	0.0%	3	10.3%	1.60	.601
	clin. rel.	10_a	83.3%	7_a	87.5%	9_a	100.0%	26	89.7%		
	total	12	100.0%	8	100.0%	9	100.0%	29	100.0%		

The cross table displays the absolute and relative frequencies of participants reporting an increase in stress or mental health problems from before to during lockdown according to our change measurement, and showing a clinically relevant or not clinically relevant questionnaire score for those stress or mental health problems during lockdown according to our state measurement. The absolute and relative frequencies are displayed for the whole sample, and separately for participants of different age groups. Furthermore, the table displays results of comparisons of frequencies for clinically relevant and not clinically relevant increases between different age groups using χ^2-tests. The alpha-level for statistical significance was set to .001.

Adapted versions of the PSQ-20 (Perceived-Stress-Questionnaire), DASS21 (Depression-Anxiety-Stress-Scales) subscales depression and anxiety, PTSS-10 (Posttraumatic-Symptom-Scale), PSWQ-PW (Penn-State-Worry-Questionnaire-Past-Week), and MK-HAI (German-modified-Health-Anxiety-Inventory) were used to measure changes in perceived stress and mental health problems during lockdown in comparison to before the COVID-19 pandemic on item level (–2 *much less than before corona*; +2 *much more than before corona*). Change indices (–1 *strong decrease* to +2 *strong increase*) were calculated for each participant and each outcome variable by averaging the change values of the total questionnaire items or the items of the respective subscale, and were grouped into three change categories (–2.00 to –1.50 *decrease*; –0.49 to +0.49 *no change*; +0.50 to +2.00 *increase*). The original questionnaires PSQ-20 total, with the subscales worries, tension, joy, and demands, DASS21 subscales depression and anxiety, PTSS-10, PSWQ-PW, and MK-HAI were used to assess the participants' state during lockdown. Clinically relevant questionnaire scores were defined according to cut-offs stated by the questionnaire authors (≥10 for DASS21 depression, ≥24 for PTSS-10, and ≥6 for DASS21 anxiety), or according to criterion c thresholds calculated with reference to clinical samples (≥54.54 for PSWQ-PW, ≥23.93 for MK-HAI, and ≥46.37 for PSQ-20 total, ≥44.84 for PSQ-20 worries, ≥45.77 for PSQ-20 tension, ≥44.66 for PSQ-20 joy, ≥40.02 for PSQ-20 demands).

clin.rel., clinically relevant. path., pathological.

$_{a/b}$: Subletters indicate significantly different proportions within one row, indicated by z-tests.
