# Supplementary material for: Individual changes in stress, depression, anxiety, pathological worry, posttraumatic stress, and health anxiety from before to during the COVID-19 pandemic in adults from Southeastern Germany
Source: BMC Psychiatry. 2022 Aug 5;22:528. doi: 10.1186/s12888-022-04148-y (PMC9354380; doi:10.1186/s12888-022-04148-y)
Supplement: Supplementary file 8 — Additional file 8: Text 1. [file 12888_2022_4148_MOESM8_ESM.pdf]

Additional Text 1. Calculating criterion c thresholds.

To calculate clinical significance thresholds for the PSQ-20 total score and subscores, the PSWQ-PW score, and the MK-HAI score, we used means and standard deviations from dysfunctional samples provided in the literature and related them to the means and standard deviations of our sample. The following values from a sample of psychosomatic patients (1) were used to calculate criterion c thresholds for the PSQ-20 scores: PSQ-20 total score: *M*=0.52, *SD*=0.18; PSQ-20 worries: *M*=0.53, *SD*=0.26; PSQ-20 tension: *M*=0.48, *SD*=0.12; PSQ-20 joy: *M*=0.37, *SD*=0.23; PSQ-20 demands: *M*=0.44, *SD*=0.16. The following values from a sample of high-worriers (2) were used to calculate a criterion c threshold for the PSWQ-PW score: *M*=60.86, *SD*=7.08. The following values from a sample with health anxiety indicated by a high Whitely Index (3) were used to calculate a criterion c threshold for the MK-HAI score: *M*=29.49, *SD*=9.12. The means and standard deviations for the respective questionnaire scores found in our sample are displayed in Table 2. The criterion c thresholds were calculated using the equation $\frac{(SD\text{crs} * M\text{oss}) + (SD\text{oss} * M\text{crs})}{SD\text{crs} + SD\text{oss}}$ (4), including the mean (M) and standard deviation (SD) values for the respective questionnaire scores from our online survey sample (oss) (see Table 2), as well as the mean and standard deviation values for the questionnaire scores from the dysfunctional samples from the literature as reported above. One criterion c threshold was calculated as clinical significance cut-off for each questionnaire score.

Literature Cited

1. Fliege H, Rose M, Arck P, Levenstein S, Klapp BF. Validierung des “Perceived Stress Questionnaire“ (PSQ) an einer deutschen Stichprobe. Diagnostica 2001; 47(3):142–52.

2. Stoeber J, Bittencourt J. Weekly assessment of worry: an adaptation of the Penn State Worry Questionnaire for monitoring changes during treatment. Behaviour Research and Therapy 1998; 36(6):645–56.

3. Bailer J, Witthöft M. Deutsches modifiziertes Health Anxiety Inventory (MK-HAI). Zusammenstellung sozialwissenschaftlicher Items und Skalen (ZIS) 2014.

4. Jacobson NS, Truax P. Clinical significance: A statistical approach to defining meaningful change in psychotherapy research. Journal of Consulting and Clinical Psychology 1991; 59(1):12–9.
